# IL-37 ameliorates myocardial fibrosis by regulating mtDNA-enriched vesicle release in diabetic cardiomyopathy mice

**DOI:** 10.1186/s12967-024-05250-3

**Published:** 2024-05-24

**Authors:** Qingyu Huang, Tongqing Chen, Jian Li, Yiming Wang, Huairui Shi, Yifei Yu, Qingwei Ji, Xiaoyan Shen, Tao Sun, Haiming Shi, Xinping Luo, Bo Jin, Yan You, Bangwei Wu

**Affiliations:** 1 Department of Cardiology, Huashan Hospital, Fudan University, Shanghai, China; 2 Department of Pharmacology & the Key Laboratory of Smart Drug Delivery, Ministry of Education, School of Pharmacy, Fudan University, Shanghai, China; 3 Department of Cardiology, Zhongshan Hospital, Fudan University, Shanghai, China; 4 Endocrinology department, Huashan Hospital, Fudan University, Shanghai, China; 5 Department of Cardiology, The People’s Hospital of Guangxi Zhuang Autonomous Region, Nanning, China

**Keywords:** Diabetic cardiomyopathy, Mitochondrial damage, Myocardial fibrosis, IL-37, MtDNA, Vesicle

## Abstract

**Background:**

Diabetic cardiomyopathy (DCM), a serious complication of diabetes, leads to structural and functional abnormalities of the heart and ultimately evolves to heart failure. IL-37 exerts a substantial influence on the regulation of inflammation and metabolism. Whether IL-37 is involved in DCM is unknown.

**Methods:**

The plasma samples were collected from healthy controls, diabetic patients and DCM patients, and the level of IL-37 and its relationship with heart function were observed. The changes in cardiac function, myocardial fibrosis and mitochondrial injury in DCM mice with or without IL-37 intervention were investigated in vivo. By an in vitro co-culture approach involving HG challenge of cardiomyocytes and fibroblasts, the interaction carried out by cardiomyocytes on fibroblast profibrotic activation was studied. Finally, the possible interactive mediator between cardiomyocytes and fibroblasts was explored, and the intervention role of IL-37 and its relevant molecular mechanisms.

**Results:**

We showed that the level of plasma IL-37 in DCM patients was upregulated compared to that in healthy controls and diabetic patients. Both recombinant IL-37 administration or inducing IL-37 expression alleviated cardiac dysfunction and myocardial fibrosis in DCM mice. Mechanically, hyperglycemia impaired mitochondria through SIRT1/AMPK/PGC1α signaling, resulting in significant cardiomyocyte apoptosis and the release of extracellular vesicles containing mtDNA. Fibroblasts then engulfed these mtDNA-enriched vesicles, thereby activating TLR9 signaling and the cGAS-STING pathway to initiate pro-fibrotic process and adverse remodeling. However, the presence of IL-37 ameliorated mitochondrial injury by preserving the activity of SIRT1-AMPK-PGC1α axis, resulting in a reduction in release of mtDNA-enriched vesicle and ultimately attenuating the progression of DCM.

**Conclusions:**

Collectively, our study demonstrates a protective role of IL-37 in DCM, offering a promising therapeutic agent for this disease.

**Graphical abstract:**

Hyperglycemia aggravates mitochondrial injury through SIRT1/AMPK/PGC1α signaling, resulting in significant cardiomyocyte apoptosis and the release of extracellular vesicles containing mtDNA in DCM mice. Fibroblasts then engulf these mtDNA-enriched vesicles, activating TLR9 signaling and the cGAS-STING pathway to initiate profibrotic process and adverse remodeling. However, both exogenous and endogenous IL-37 ameliorate mitochondrial injury by preserving the activity of SIRT1-AMPK-PGC1α axis, and reducing the release of mtDNA-enriched vesicles, which attenuates the progression of DCM

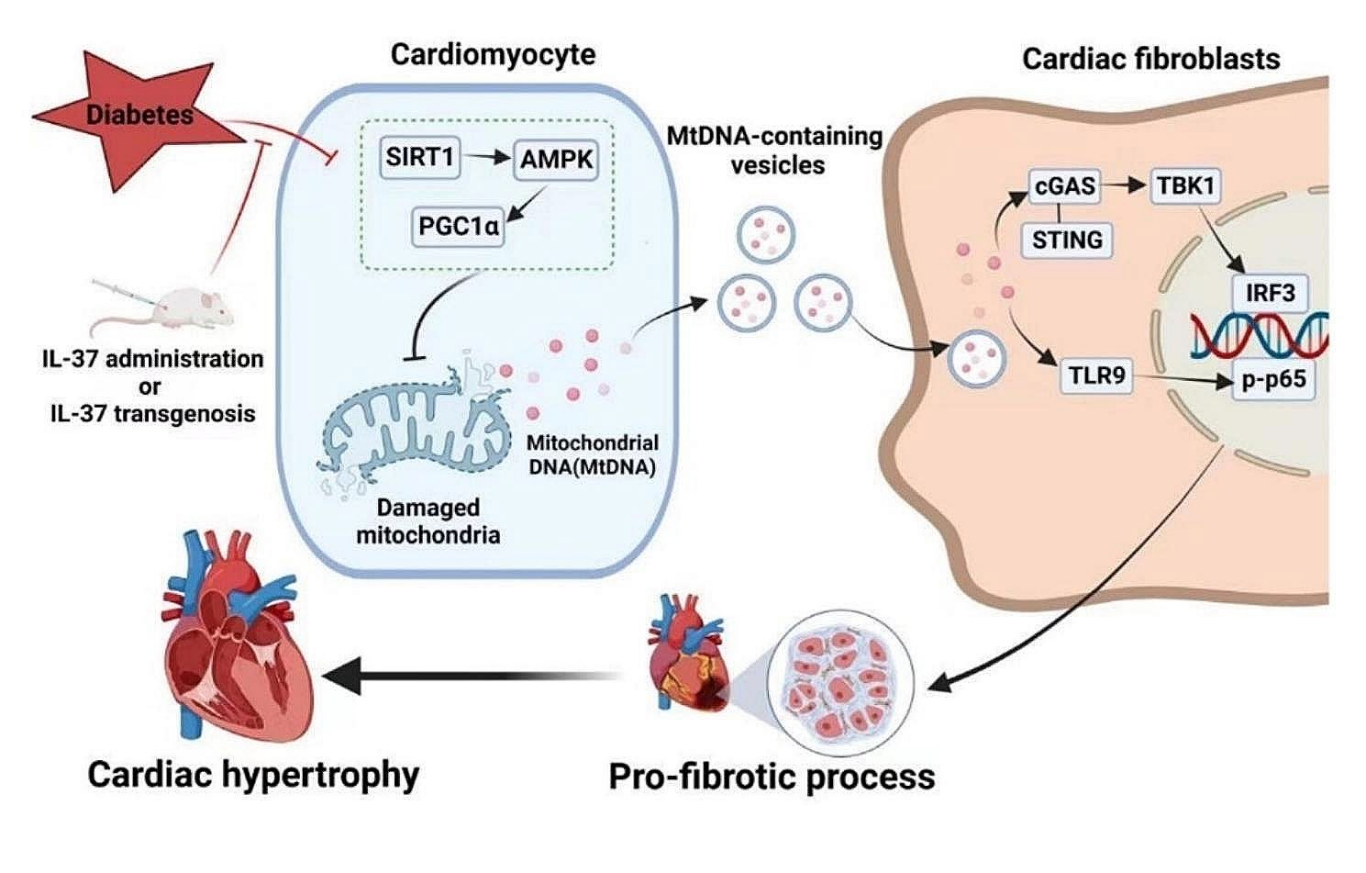

**Supplementary Information:**

The online version contains supplementary material available at 10.1186/s12967-024-05250-3.

## Introduction

Approximately 22% diabetic patients develop myocardial dysfunction, known as diabetic cardiomyopathy (DCM), even in the absence of traditional cardiac risk factors such as coronary artery disease, uncontrolled hypertension, and congenital heart disease [1, 2]. The characteristic pathological alterations in DCM include oxidative stress, fibrosis, hypertrophy, cardiac diastolic dysfunction and eventually systolic heart failure [2, 3]. However, treatment options for DCM are limited and the underlying mechanisms remain incompletely understood [4].

IL-37, formerly known as IL-1F7, is a novel anti-inflammatory cytokine in the IL-1 ligand family, but the only IL-1 family member that is not found in mice [5].IL-37 plays a pivotal role in both innate and adaptive immunity, and has been implicated in the pathogenesis of various inflammatory diseases, such as cardiovascular diseases, inflammatory bowel disease, asthma, autoimmune disease and multiple sclerosis and so on [6, 7]. Recent studies have also shown that IL-37 plays a role in tumorigenesis, angiogenesis, autophagy and insulin sensitivity [8, 9]. Mice treated with IL-37 could induce remarkable metabolic changes with higher levels of muscle AMP-activated protein kinase (AMPK), greater rates of oxygen consumption, and increased oxidative phosphorylation [10]. Besides, we and other researchers have shown that IL-37 could protect cardiomyocytes and endothelial cells from apoptosis by alleviating ROS stress, suggesting its possible involvement in the pathophysiology of hyperglycemia-induced cardiomyopathy [7, 11, 12].

Mitochondria play crucial roles in cellular metabolism, calcium homeostasis, and immune response [13]. Emerging evidence suggests that mitochondrial dysfunction contributes to the onset and progression of DCM [14–16]. Impaired glucose homeostasis, oxidative stress, and imbalanced mitochondrial dynamics in diabetic heart all result in mitochondrial dysfunction [4, 14, 17]. The damaged mitochondria subsequently release damage-associated molecular patterns (DAMPs), including mitochondrial DNA (mtDNA), N-formyl peptides and lipids, which contribute to the development of various diseases, such as cardiovascular disease and neurological disorders [18]. Interestingly, studies have shown a significant increase in mtDNA damage and release from diabetic hearts [19–21]. Furthermore, cardiomyocyte-derived mtDNA is sufficient to cause severe myocarditis and dilated cardiomyopathy [22]. However, the potential role of mtDNA in DCM remains unexplored.

Here, we showed the upregulated levels of plasma IL-37 in DCM patients, and demonstrated the protective role of IL-37 on cardiac dysfunction and myocardial fibrosis in DCM mice. Mechanistically, the cardioprotective effect of IL-37 in DCM was through the maintenance of SIRT1-AMPK-PGC1α axis, thereby preventing mitochondrial dysfunction and the release of mtDNA-containing vesicles in cardiomyocytes. As fibroblasts engulfed these mtDNA-enriched vesicles, activating TLR9 signaling and the cGAS-STING pathway, ultimately resulting in the fibrosis of diabetic hearts.

## Method and materials

### Human blood sample collection

Human blood sample collection was approved by the Human Ethics Committee, HuaShan Hospital, Fudan University (2016 Trial No. 395), and the study was conducted in accordance with the principles of Good Clinical Practice and the Declaration of Helsinki. Informed consent was obtained from participants or their guardians. Consecutive participants who met the inclusion criteria and were hospitalized between May 2020, to January 2021 in Huashan hospital were enrolled. Fasting plasma samples were collected from the initial admission blood draws of those patients who were diagnosed as diabetic cardiomyopathy. Specified inclusion criteria of DCM are as following [23, 24]: (1) presence of diabetes mellitus; (2) diagnosis of systolic and diastolic left ventricular dysfunction with echocardiography; (3) absence of coronary disease, hypertension or significant valvular disease.

### Animal care and establishment of diabetic models

All animal experiments were approved by the Institutional Animal Care and Use Committee at Fudan University(201,802,049 S), and carried out in accordance with the National Institutes of Health guidelines. Heterozygous hIL-37b conditional knock-in mice with a C57BL/6 background, containing the “CAG promoter loxP-stop-loxP- hIL37b CDS-polyA” at the H11 site of genome, was generated using CRISPR/Cas9 technology (GemPharmatech Co. Ltd, Jiangsu, China), and then bred to obtain homozygous hIL-37b conditional knock-in (H11 CKI/CKI) mice. At this moment, the hIL-37b sequence cannot be transcribed, due to the “loxP-stop-loxP” was inserted to split the CAG promoter and the hIL-37b CDS. To generate a cardiomyocyte specific hIL-37b overexpression mouse (Myh6-CreER-IL-37 mice, abbreviated as IL-37-Tg mice), the homozygous hIL-37b conditional knock-in (H11 CKI/CKI) mice were crossed with Myh6-CreER mice (Shanghai Model Organisms Center, Inc). For inducible IL-37 expression, tamoxifen (75 mg/Kg body weight) was injected intraperitoneally into IL-37-Tg mice for five consecutive days. Each mouse strain was genotyped by PCR using DNA extracted from mouse tail tissues. Throughout the study, mice were provided ad libitum access to water and food while being housed under controlled temperature conditions (18–24 °C) and a reverse 12-hour light-dark cycle. Insulin deficiency was induced by a single intraperitoneal injection of 100 mg/kg streptozotocin (STZ, Sigma, St. Louis, MO, USA) in sodium citrate buffer (100 mmol/L, pH 4.5). Fasting blood glucose was measured 5 days after injection and only mice with blood glucose levels higher than 13.9 mmol/L were defined as diabetic. Diabetic cardiomyopathy (DCM) was induced by feeding them on high-fat diet for 12 consecutive weeks [25]. Recombinant hIL-37b (RhIL-37) was administered intraperitoneally twice a week at a dose of 1 µg per mouse fed high-fat diet synchronously (R&D, Minneapolis, USA) [10]. After cardiac function was assessed, the animals were anesthetized by inhalation of 2% isoflurane in oxygen and sacrificed via cervical dislocation, and the hearts were isolated for follow-up experiments.

### Echocardiography

A Visual Sonics Vevo 770 ultrasound machine equipped with a 30 MHz phase array linear transducer was used (Visual Sonics Inc, Toronto, Ontario, Canada). The mice were anaesthetized with 2% isoflurane. M-mode images were used to measure LV fractional shortening (FS, %), LV ejection fraction (EF, %), LV end-diastolic dimension (LVEDD, mm), and LV end-diastolic volume (LVEDV, µL), which were acquired by a technician who was blind to the treatment groups.

### Assessment of cardiac fibrosis

Isolated mouse hearts were fixed with 4% paraformaldehyde and subsequently embedded in paraffin before being sectioned. The paraffin-embedded sections then were deparaffinized and stained by a Masson’s Trichrome Stain Kit or a Picro Sirius Red Stain Kit (PolySciences, Inc). Sections were scanned with a Nikon iEclipse microscope and quantified using Image J software.

### Transmission electron microscopy

Transmission electron microscopy (TEM) for morphological analysis was performed as described previously [26]. Heart tissue was fixed in 2.5% glutaraldehyde in phosphate buffer overnight at 4 °C. After sample preparation, 90–100 nm thick sections were mounted onto a 200-mesh copper grid and imaged with a JEOL 1200EX electron microscope.

### Adult cardiomyocyte (CM) isolation

Adult cardiomyocytes were isolated from the ventricles of wild-type (C57BL/6J), and IL-37-Tg mice as described previously with some adjustments [27]. Briefly, male mice aged 8 to 12 weeks were anesthetized by inhalation of 2% isoflurane in oxygen and sacrificed via cervical dislocation. After exposing the heart, then cutting the descending aorta, and 7 ml of EDTA buffer was immediately injected into the right ventricle for cardiac perfusion. The heart was then transferred to a 60-mm dish with fresh EDTA buffer. To achieve heart digestion, a sequential injection of EDTA buffer, perfusion buffer, and collagenase buffer was administered into the left ventricle. Subsequently, the heart was dissected and gently pulled into 1-mm pieces to release cells. Cellular separation was completed by gentle grinding, before inhibiting the enzymatic activity by stop buffer. The cell suspension was filtered through a 100-µm filter and underwent 3 sequential rounds of gravity settling to obtain a highly pure myocyte fraction. In that progress, 3 intermediate calcium reintroduction buffers were used to gradually restore the calcium concentration to physiological levels. Finally, the cardiac myocytes were resuspended in prewarmed plating media and plated onto laminin(5 µg/mL)-precoated plates in a humidified tissue culture incubator (37 °C, 5% CO_2_). After 1 h and every 48 h thereafter, the media was replaced with fresh, prewarmed culture media. To silence SIRT1 and PGC1α, CMs were transfected with either non-targeting siRNA (NC) or siRNA targeting SIRT1 or PGC1α for 48 h. Subsequently, the cells were stimulated with 25 mM glucose (HG) for 24 h to mimic the in vivo environment. To investigate whether the inhibitory effect of IL-37 on fibrosis was dependent on its ability to regulate cardiomyocyte paracrine pathway, IL-37-Tg cardiomyocytes or WT cardiomyocytes treated with or without IL-37(1ng/ml)were exposed to 10µM vesicle inhibitor GW4869 (Sigma-Aldrich, St. Louis, MO, USA) for 24 h. Subsequently, they were co-cultured with fibroblasts or their supernatant was collected and used to stimulate fibroblasts for another 24 h.

### Cardiac fibroblast (CF) isolation

To isolate mouse adult cardiac fibroblasts (CF), the hearts were minced into small pieces smaller than 1 mm^3^ and then were washed in Ca^2+^-free PBS and digested with 0.04% trypsin and 0.05% collagenase IV at 37 °C for 5 min. The resulting supernatant was collected, and an equal volume of fresh medium was added to terminate the digestion process. Following digestion, fibroblasts were isolated by filtration through a 40 μm nylon mesh and subsequently centrifuged at 300 g for 5 min. The pellet was resuspended in 5 mL culture media before being spread onto a dish. CFs were co-cultured with WT or IL-37-Tg CM, either in the presence or absence of 5 µM ODN1826 (InvivoGen, San Diego, CA) or 5 µg/ml 2’,3’-cGAMP (InvivoGen, San Diego, CA), for a duration of 24 h. To further confirm that the inhibitory effect of IL-37 on fibrosis involved reduced release of vesicles by cardiomyocytes, an endocytosis assay was conducted. In brief, CFs were treated with 10µM chlorpromazine (CPZ, Sigma), an endocytosis inhibitor, for 30 min prior to co-culture with either WT or IL-37-Tg CM in the presence of HG for 24 h or stimulation with vesicles derived from WT or IL-37-Tg CM for 24 h. Subsequently, western blotting and immunofluorescence analysis were performed to assess the fibrotic process and endocytosis levels.

### Analysis of apoptosis

HG-treated or untreated cardiomyocytes were subjected to si-SIRT, compound C (10µM), or si-PGC1α treatment. The apoptotic rates of cardiomyocytes were assessed with a one-step TUNEL kit as per the manufacturer’s instruction (Beyotime, Shanghai, China), followed by flow cytometry analysis. The apoptotic rates in the heart were evaluated through fluorescence microscopy detection. The number of TUNEL-positive cells was quantified in five randomly selected fields, and the ratio of TUNEL-positive cells to DAPI-positive cells was calculated.

### Assessment of mitochondrial membrane potential

The mitochondrial membrane potential (ΔΨm) was analyzed using a JC-1 kit from Invitrogen (Thermo Fisher Scientific, Waltham, MA) and detected by fluorescence microscopy.

### Western blot analysis

The total lysates from heart tissue or whole cell lysates were immunoblotted and probed with primary antibodies as described previously [11]. Densitometry was performed using Image Pro Plus software (Media Cybernetics, Silver Spring, MD, USA). The antibodies and dilutions used in this study are shown in Supplementary Table 2.

### Real-time qPCR for mitochondrial DNA

Extracellular vesicles were isolated by ultracentrifugation as described previously [28], and vesicle concentration was determined by nanoparticle tracking analysis (NTA). The mtDNA of vesicles in culture supernatant from WT and IL-37-Tg CM, plasma of patients and mice, myocardial tissue of mice was extracted and purified separately using the QIAamp DNA Blood Mini Kit or DNeasy Blood & Tissue Kit (Qiagen) as described previously [29, 30]. MtDNA content was assessed by using real time PCR (RT-PCR). The primer sequences for mtDNA and mRNA of other genes tested in this paper are shown in Supplementary Table 3.

### Statistical analysis

Statistical analyses were performed using the SPSS 23.0 software or Graph Pad Prism 8.0. The data are presented as mean ± SEM, and the difference in means between two groups or multiple groups was assessed by unpaired Student t-test or one-way ANOVA, followed by Bonferroni post hoc test. A P values less than 0.05 were considered statistically significant.

## Results

### Plasma IL-37 level was upregulated and positively correlated with the severity of heart dysfunction in DCM patients

**Fig. 1:**
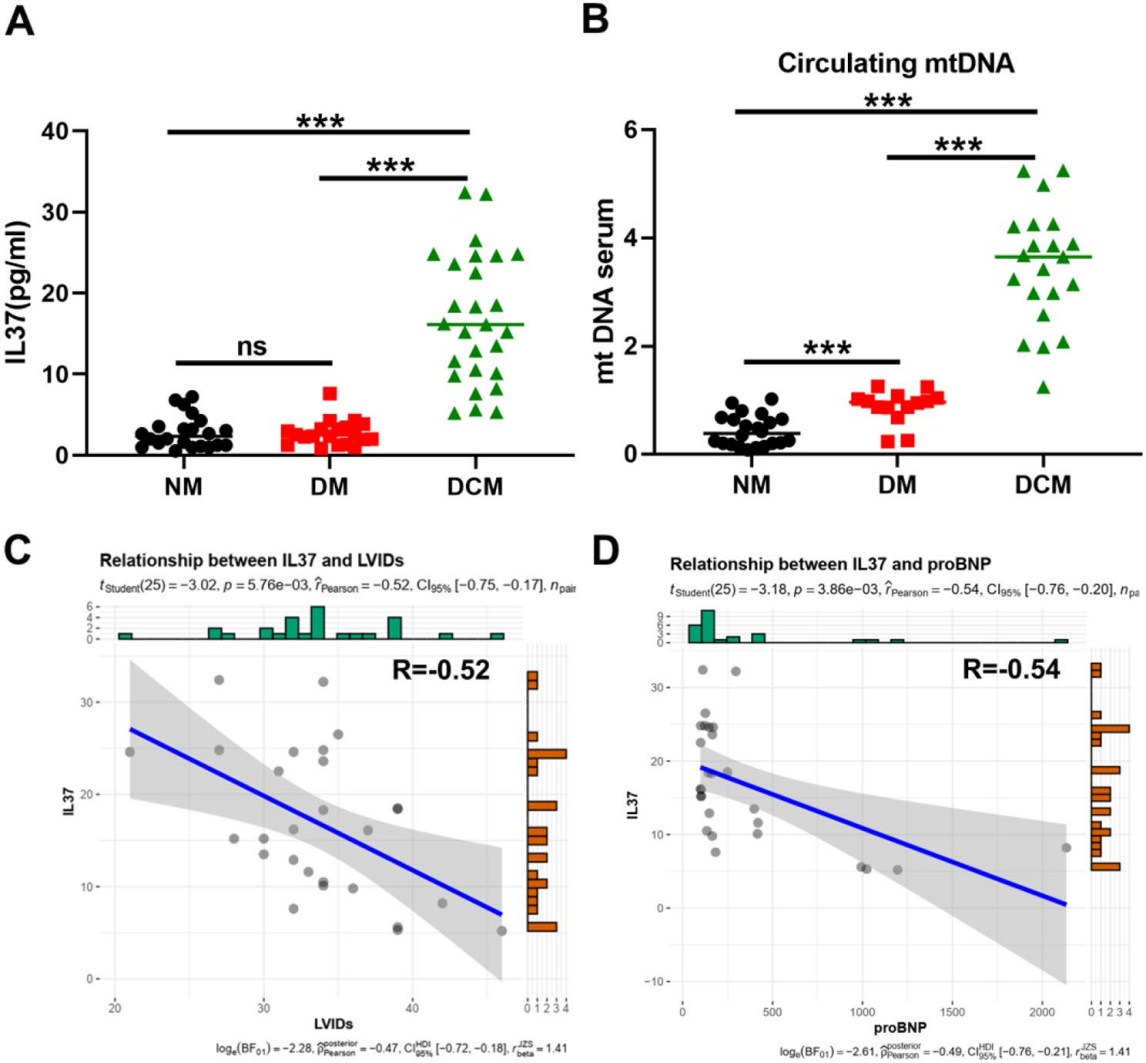
The plasma IL-37 level was increased in diabetic patients. (**A**) Serum IL-37 levels in healthy individuals, and diabetic patients with or without cardiomyopathy. (**B**) Circulating mtDNA level in plasma samples in diabetic patients and healthy controls. (**C**) The correlation between IL-37 and LV internal dimension index diastole (LVIDIs) was analyzed by Spearman. (**D**) The correlation analysis by Spearman between IL-37 and N-terminal fragment brain natriuretic peptides (pro-BNP) in DCM patients. ***P< 0.001, data were analyzed by one way ANOVA followed by Bonferroni post hoc test

cardiomyopathy, the plasma samples were collected from healthy controls, diabetic patients and cardiovascular patients with diabetes at Huashan Hospital in Shanghai, China (Supplementary Table 1). As shown in Fig. 1A, IL-37 exhibited a slight elevation in plasma samples obtained from diabetic patients and healthy controls. Notably, DCM patients demonstrated the highest levels of IL-37. MtDNA can be released into the circulation from stressed cells, which reflects systemic mitochondrial damage and inflammation response [29]. We next detected the circulating mtDNA level in plasma samples obtained from diabetic patients and healthy controls. Interestingly, DCM patients showed the highest level of mtDNA compared with diabetic patients and healthy controls (Fig. 1B). A negative correlation was revealed between the severity of DCM diagnosed clinically and the level of IL-37 (Fig. 1C). Instead, the heart function marker pro-BNP exhibited a negative correlation with IL-37 expression (Fig. 1D), providing further evidence of the potential role of IL-37 in the development of DCM.

### Recombinant IL-37 administration alleviated HFD-induced cardiac dysfunction and cardiac hypertrophy in diabetic mice

**Fig. 2:**
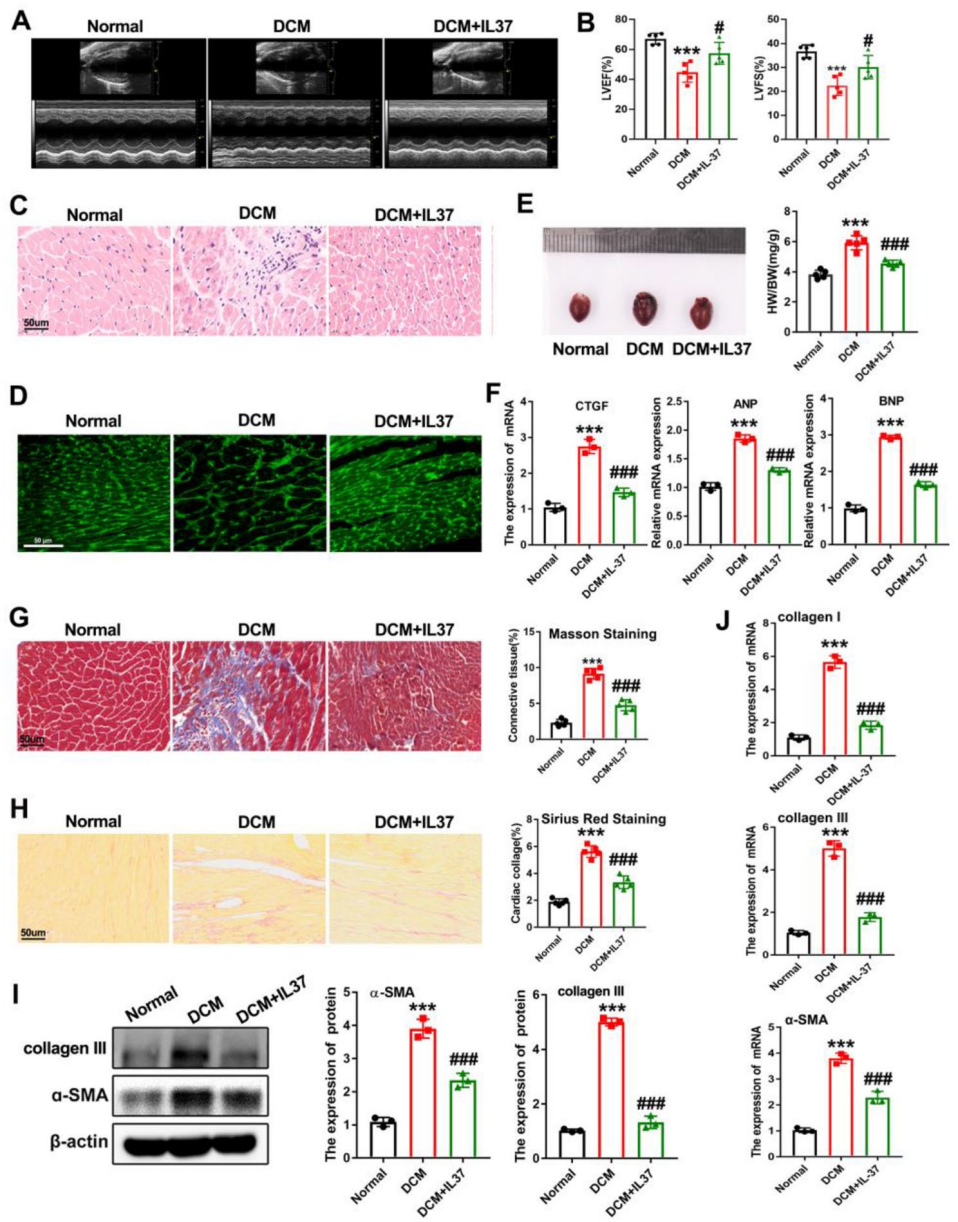
IL-37 administration alleviated high-fat diet (HFD)-induced cardiac dysfunction and cardiac hypertrophy in mice. (**A**) Representative M-mode echocardiograms of left ventricle. (**B**) Quantitative analyses of ejection fraction (EF) and fractional shortening (FS). (**C**) Hematoxylin-eosin (HE) staining was performed. Scale bar, 50 μm. (**D**) Representative images of cardiomyocyte size obtained using wheat germ agglutinin (WGA) staining. Scale bar, 50 μm.(**E**) Representative illustration of whole heart and heart-to-body weight (right) ratio from mice treated with or without IL-37. (**F**) The expression of myocardial hypertrophy markers of ANP, BNP and CTGF in DCM mice were detected by qRT-PCR. (**G**) Representative images of Masson trichrome staining of hearts and quantitative analysis of interstitial fibrosis are presented. Scale bar, 50 μm. (**H**) Representative images of cardiac tissue sections stained with picrosirius red and the quantification are displayed in the right panel. Scale bar, 50 μm. (**I**) The protein levels of collagen III and α-SMA were detected by western blot. (**J**) Real-time PCR analysis was performed to determine the mRNA expression levels of collagen I, collagen III, and α-SMA. Values are means ± SEM; *P< 0.05, **P< 0.01, ***P< 0.001 vs. normal group; ^#^*P*< 0.05, ^##^*P*< 0.01, ^###^*P*< 0.001 vs. DCM group. (*n* = 6 in each group, data were analyzed by one way ANOVA followed by Bonferroni post hoc test.)

body weight along with increased blood glucose (Supplementary Figure S1A-B), while no difference was found in cardiac function (Supplementary Figure S1C). HFD-fed diabetic mice had higher serum total cholesterol (TC), triacylglycerol (TG) and insulin levels, hyperglycemia, and gained weight faster than diabetic mice fed normal diet (Supplementary Figure S2A-E). Moreover, a deterioration of the systolic and diastolic functions of the left ventricular, and the reduction of ejection fraction (EF) were observed in the HFD-fed diabetic mice, indicating that HFD-fed diabetic mice developed a DCM phenotype (Supplementary Figure S2F). Interestingly, when treated the DCM mice with recombinant IL-37, the damaged cardiac function was dramatically reversed, but not the body weight or blood glucose (Fig. 2A-B, Supplementary Figure S2D-E). In addition, the cardiac hypertrophic features in DCM mice including increased heart weights, enlarged cardiomyocytes and upregulated myocardial hypertrophic markers were significantly inhibited by IL-37 administration, as evidenced by decreased heart cross-sectional area, reduced cardiomyocyte size and downregulated mRNA levels of CTGF, ANP and BNP (Fig. 2C-F). Both Masson’s trichrome staining and Sirius Red staining revealed that IL-37-treated DCM mice had lower collagen deposition than untreated DCM mice (Fig. 2G-H). As shown in Fig. 2I, the protein levels of collagen III and α-SMA in the hearts of DCM mice were reduced in the presence of IL-37. Accordingly, the upregulated mRNA levels of α-SMA, collagen type I α1 (Collagen I) and collagen type III in DCM mice were also notably suppressed by IL-37 (Fig. 2J). The above results demonstrated that cardiac dysfunction and abnormal ventricular remodeling in diabetic condition could be alleviated by IL-37.

### Cardiomyocyte specific IL-37 expression prevented cardiomyopathy in diabetic mice

**Fig. 3:**
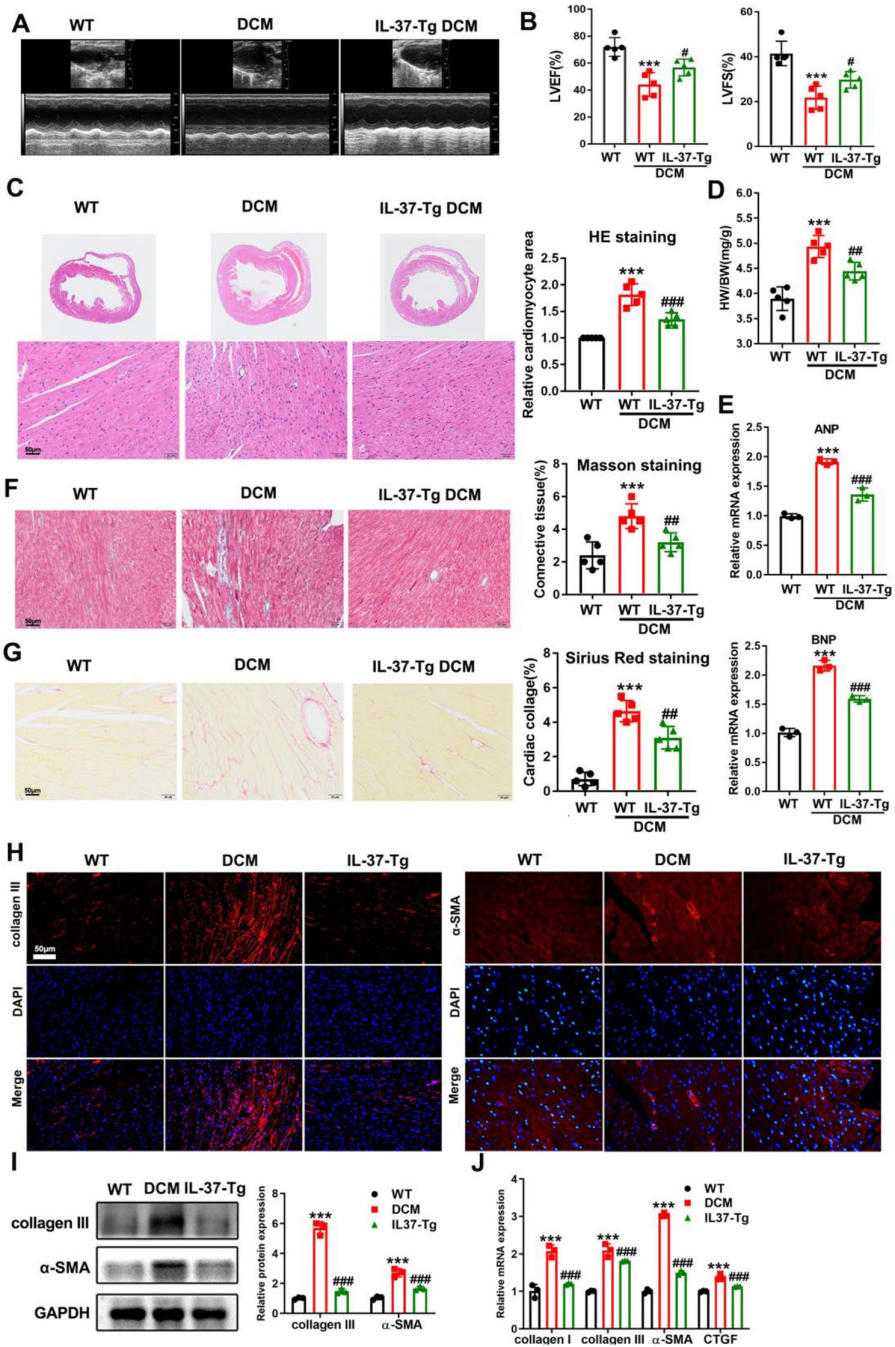
Cardiomyocyte-specific IL-37 expression prevented cardiomyopathy in diabetic mice. (**A**) Representative M mode tracing of echocardiographs. (**B**) FS and EF were evaluated by echocardiography. (**C**) The gross morphology of hearts stained by HE staining. Scale bar, 50 μm. (**D**) The heart-to-body weight ratio. (**E**) The mRNA levels of ANP and BNP detected by qRT-PCR. (**F** and **G**) Representative images of Masson trichrome staining and picrosirius red staining of hearts and quantitative analysis of interstitial fibrosis are presented. (**H**) Immunohistochemistry analysis was performed to detect the expression of collagen III and α-SMA. (**I**) The protein levels of collagen III and α-SMA were detected by western blot. (**J**) Real-time PCR analysis was performed to determine the mRNA expression levels of collagen I, collagen III, α-SMA and CTGF. Values are means ± SEM; *P< 0.05, **P< 0.01, ***P< 0.001vs normal group; ^#^*P*< 0.05, ^##^*P*< 0.01, ^###^*P*< 0.001 vs. DCM group. (*n* = 6 in each group, data were analyzed by one way ANOVA followed by Bonferroni post hoc test.)

To further confirm which cells respond to IL-37, we first generated cardiomyocyte-specific IL-37-transgenic (IL-37-Tg) mice. These mice had higher IL-37 mRNA only in whole heart extracts (Supplementary Figure S3A). RT-PCR verified high expression of IL-37 in isolated primary cardiomyocytes (Supplementary Figure S3B). As shown in Supplementary Figure S3C-D, IL-37 had no significant effects on serum TC and insulin levels in DCM mice. Echocardiography data showed that IL-37-Tg mice were protected from cardiac dysfunction (Fig. 3A-B). H&E staining and the ratio of heart to weight revealed that the increased myocardial mass in HFD-fed diabetic hearts was ameliorated after expressing IL-37 (Fig. 3C-D). Similarly, the upregulation of cardiac hypertrophic markers including ANP and BNP was suppressed by IL-37 expression (Fig. 3E). Compared to WT DCM mice, a much lower level of collagen fiber accumulation was observed in IL-37-Tg DCM mice, as detected by Masson staining and Sirius red staining (Fig. 3F-G). The attenuated expression of a-SMA and collagen III in IL-37-Tg mice further supported that cardiomyocyte-specific IL-37 expression could suppress cardiac fibrosis in DCM mice (Fig. 3H-J). Together, the above results indicated that the reduced perivascular fibrosis in IL-37-Tg mice might contribute to improved cardiac function, which was not accompanied by altered systemic glucose management.

### Diabetes-triggered destruction of mitochondrial morphology and function was suspended by IL-37

**Fig. 4:**
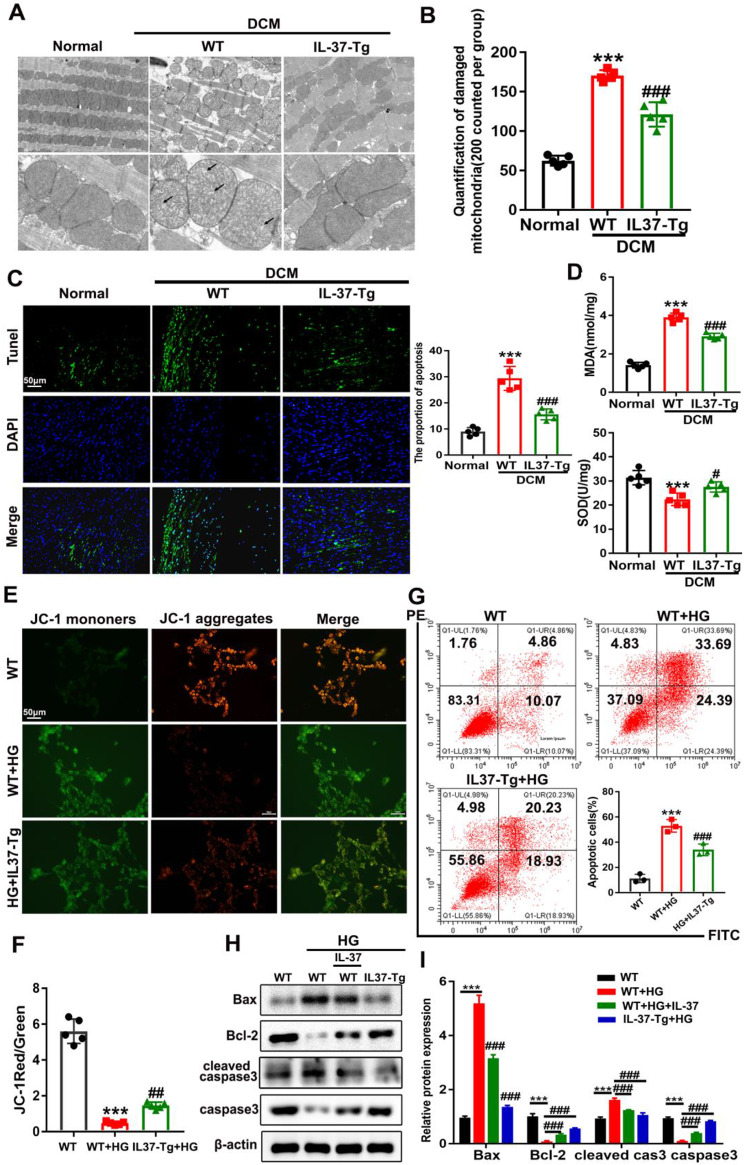
IL-37 Prevents Diabetes-triggered Destruction of Mitochondrial Morphology and Function. (**A**) Representative transmission electron microscopy (EM) images of heart sections. Scale bar, 0.5 μm Magnification ×400. (**B**) The proportion of morphologically impaired mitochondria to intact ones. (**C**) Representative photomicrographs of TUNEL-stained and DAPI-stained heart sections, accompanied by a bar graph (on the right) illustrating the proportion of apoptotic cells. Scale bar, 50 μm. (**D**) The levels of SOD and MDA in cardiac tissue homogenates were detected using commercially available kits. (**E** and **F**) Mitochondrial membrane potential was assessed by JC-1 staining and quantified through the JC-1 shift ratio. Red fluorescence represents JC-1 aggregation, while green fluorescence indicates JC-1 monomer that enters into the cytosol after mitochondrial membrane depolarization. Scale bar, 50 μm. (**G**) Flow cytometry analysis of apoptosis by annexin V and PI staining. (**H** and **I**) The expression levels of apoptosis-related proteins, including BAX, Bcl-2, cleaved caspase 3 (cas3) and caspase 3. Values are means ± SEM; *P< 0.05, **P< 0.01, ***P< 0.001vs non-treated WT group; ^#^*P*< 0.05, ^##^*P*< 0.01, ^###^*P*< 0.001 vs. HG-treated WT group. At least 3 independent experiments were conducted. (*n* = 6 in each group(A-D), *n* = 3 in each group at least(E-I), data were analyzed by one way ANOVA followed by Bonferroni post hoc test.)

Mitochondrial disfunction plays a pivotal role in the development of DCM [31]. To investigate how IL-37 exerted the protective effects against cardiac dysfunction and fibrosis in DCM mice, we then performed high-resolution electron microscopy analysis to examine mitochondrial morphology in diabetic IL-37-Tg mice and their WT littermates. As shown in Supplementary Figure S4A-B, although HFD-fed diabetic heart tissues had mildly lower mitochondrial density than the hearts of lean mice, no difference was observed between IL-37-Tg mice and their WT littermates, indicating that IL-37 has no effect on mitochondrial biogenesis or turnover in diabetic hearts. Interestingly, WT DCM mice showed more severe damage in mitochondrial morphology with inflation, warped membranes, irregularities, and the absence of cristae than IL-37-Tg DCM mice (Fig. 4A-B). Multiple studies have demonstrated that mitochondrial disfunction could promote oxidative stress and cardiomyocyte apoptosis, through which it facilitates the progression of DCM. As expected, there were reduced cardiomyocyte apoptosis and oxidative stress in IL-37-Tg DCM mice compared with WT DCM mice (Fig. 4C-D). These results suggested that IL-37 mitigated diabetes-induced mitochondrial dysfunction followed by a decrease in oxidative stress and apoptosis.

To support our conclusion, cardiomyocytes were isolated from adult IL-37-Tg mice and WT mice and treated with 25 mM glucose (HG) for 24 h to mimic an in vivo high glucose environment. JC-1 staining showed decreased mitochondrial membrane potential in WT cardiomyocytes, indicating that HG induced mitochondrial dysfunction in cardiomyocytes (Fig. 4E-F). However, this downward trend was ameliorated by inducing IL-37 expression. Flow cytometry analysis also showed that IL-37 could reduce HG-induced apoptosis in cardiomyocytes (Fig. 4G). Consistent with the in vivo findings, IL-37 expression or treatment suppressed the increased levels of apoptosis-related proteins (BAX, Caspase-3 and cleaved Caspase-3) and attenuated the decreased level of Bcl-2 induced by HG treatment in cardiomyocytes (Fig. 4H-I). All these results indicated that IL-37 might exert a protective effect on cardiac structure and function in DCM mice by alleviating mitochondrial damage.

### IL-37-mediated SIRT1/AMPK/PGC-1α Axis accounted for the reduced mitochondrial damage and apoptosis

**Fig. 5:**
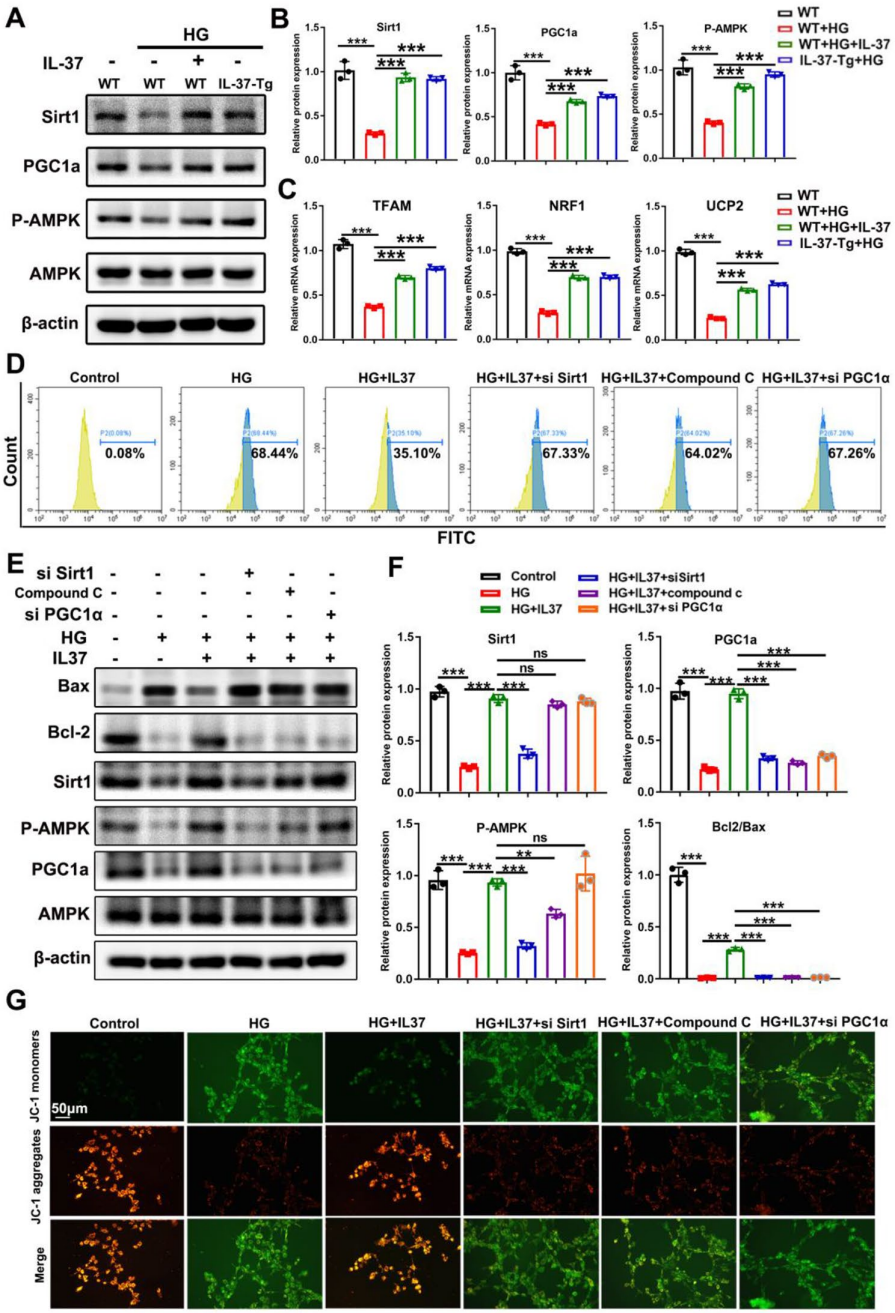
IL-37-mediated SIRT1/AMPK/PGC-1α Axis Accounts Reduced Mitochondrial Damage and Apoptosis. (**A** and **B**) The protein levels of SIRT1, PGC1α, p-AMPK and AMPK were assessed in cardiomyocytes treated as indicated using western blot analysis. (**C**) The transcriptional activation of genes targeted by PGC1α. (**D**) Flow cytometry analysis was performed to evaluate oxidative stress in cardiomyocytes treated with si-SIRT1, compound C or si-PGC1α in the presence or absence of IL-37 and HG. (**E** and **F**) The apoptotic signals in cardiomyocytes treated as described in D were determined by western blot and quantified using Image J. CF: fibroblast; CMs: cardiomyocytes. (**G**) Mitochondrial membrane potential in cardiomyocytes treated as described in D was assessed by JC-1 staining. Scale bar, 0.5 μm. Values are means ± SEM; *P< 0.05, **P< 0.01, ***P< 0.001. (*n* = 3 in each group at least, data were analyzed by one way ANOVA followed by Bonferroni post hoc test.)

Here we need to explore the underlying molecular mechanisms by which IL-37 exerts on mitochondrial protection against HG-induced cardiomyopathy. Considering that both AMPK and SIRT1 are involved in maintaining mitochondrial homeostasis under various perturbations of cardiomyocytes, we measured their expression in HG-treated cardiomyocytes and found that the protein levels of p-AMPK (activated form of AMPK) and SIRT1 were reduced after HG treatment (Fig. 5A and B), which was consistent with the results of decreased protein levels in the myocardium of DCM mice (Supplementary Fig. 5A-B). In the presence of IL-37, the downregulation of p-AMPK and SIRT1 was arrested (Supplementary Fig. 5A-B). Moreover, the enhanced expression of PGC1α, a target for both AMPK and SIRT1, and its downstream molecules was further confirmed in both in vivo and in vitro experiments (Supplementary Fig. 5AB, Fig. 5A-C).

To confirm that IL-37-induced mitochondrial protection was SIRT/AMPK/PGC1α dependent, we treated WT cardiomyocytes with the AMPK inhibitor Compound C (10µM), SIRT1-targeted siRNA or PGC1α-targeted siRNA in the absence or presence of HG or IL-37. The efficiency of siRNA was confirmed by both qPCR and western blot (Supplementary Figure S6A-B). The lower levels of ROS and apoptosis-related proteins observed in IL-37-treated cells were abolished by Compound C, SIRT1 silencing and PGC1α silencing (Fig. 5D-G). Interestingly, we found that Compound C did not affect the upregulation of SIRT1 in IL-37-treated cardiomyocytes (Fig. 5E-F). However, silencing SIRT1 abolished the upregulation of p-AMPK-triggered by IL-37. Accordantly, the upregulation of PGC1α-induced by IL-37 was suppressed by both SIRT1 silencing and Compound C treatment. Whether the mitochondrial membrane potential-stabilizing activity of IL-37 could be restrained by Compound C, SIRT1 silencing or PGC1α silencing were analyzed. As shown in Fig. 5G, any of them significantly disturbed the stabilizing effect of IL-37 on mitochondrial membrane potential. All the above results suggested that the mitochondrial protective effect of IL-37 in cardiomyocytes is related to SIRT1-mediated activation of AMPK followed by increased PGC-1α expression, through which IL-37 possesses anti-cardiac hypertrophy property.

### Damaged cardiomyocytes released vesicles to activate profibrotic signaling in cardiac fibroblasts, which can be ameliorated by IL-37

**Fig. 6:**
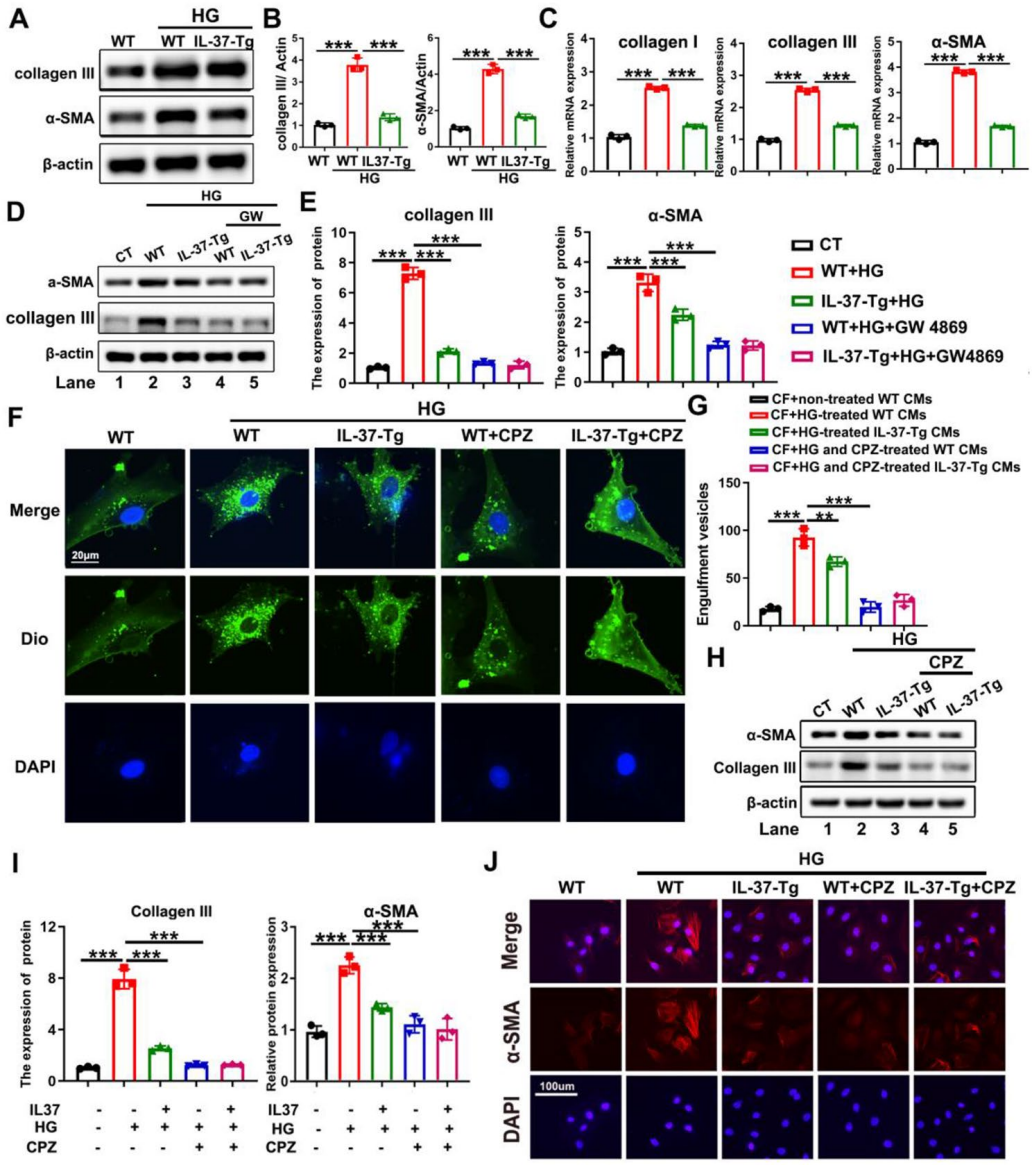
Damaged cardiomyocytes Release Vesicles to Activate pro-fibrotic signaling in Cardiac Fibroblasts. (**A** and **B**) Adult WT and IL-37-Tg cardiomyocytes treated with or without HG were co-cultured with primary cardiac fibroblasts. The protein levels of collagen III and α-SMA were detected by western blot. (**C**) Fibroblasts were subjected to the treatment described in A, and RT-qPCR was applied to detect the expression of collagen I, collagen III and α-SMA. (**D** and **E**) Adult WT and IL-37-Tg cardiomyocytes were exposed to HG or GW4869 (GW), and the culture supernatant was collected and applied to primary cardiac fibroblasts respectively. The protein levels of collagen III and α-SMA in fibroblasts were subsequently detected using western blot analysis. Lane 1 represents fibroblasts treated with culture supernatant from untreated WT cardiomyocytes (CT); Lane 2 represents fibroblasts treated with culture supernatant from HG-treated WT cardiomyocytes (WT+HG); Lane 3 represents fibroblasts treated with culture supernatant from HG-treated IL-37-Tg cardiomyocytes (IL-37-Tg+HG); Lane 4 represents fibroblasts treated with culture supernatant from both HG and GW4869-treated WT cardiomyocytes (WT+HG+GW4869); Lane 5 represents fibroblasts treated with culture supernatant from both HG and GW4869-treated IL-37-Tg cardiomyocytes (IL-37-Tg+HG+GW4869). (**F** and **G**) The internalization of vesicles derived from WT and IL-37-Tg cardiomyocytes by fibroblasts pre-treated with or without CPZ was visualized (**F**), and the average number of engulfed vesicles per cell was quantified (**G**). Scale bar, 20 μm. (**H** and **I**) The protein levels of collagen III and α-SMA in fibroblasts treated as described in F were analyzed using western blotting. The induction of IL-37 expression in IL-37-Tg cardiomyocytes is mediated by tamoxifen (TAM). (**J**) Representative microphotographs of α-SMA staining in fibroblasts treated as described in F were obtained. Scale bar, 100 μm. Values are means ± SEM; *P< 0.05, **P< 0.01, ***P< 0.001. (*n* = 3 in each group at least, data were analyzed by one way ANOVA followed by Bonferroni post hoc test.)

Since collagen deposition and cardiomyocyte damage were significantly alleviated by cardiomyocyte-specific IL-37 expression in DCM mice, we suspected that IL-37 might protect cardiomyocytes from releasing extracellular vesicles containing a certain toxic molecule to activate fibroblasts. To prove our hypothesis, adult WT and IL-37-Tg cardiomyocytes treated with or without HG were co-cultured with primary cardiac fibroblasts. As shown in Fig. 6A and B, the protein levels of α-SMA and collagen III in fibroblasts co-cultured with HG-treated WT cardiomyocytes were significantly higher than that in fibroblasts co-cultured with HG-treated IL-37-Tg cardiomyocytes. This result was further supported by RT-qPCR analysis (Fig. 6C). Next, primary fibroblasts were incubated with the culture supernatant from HG or GW4869 (an exosome secretion inhibitor)-treated WT and IL-37-Tg cardiomyocytes and the protein levels of α-SMA and collagen III were detected. As expected, the presence of GW4869 in HG-treated WT cardiomyocytes mimicked IL-37-induced downregulation of α-SMA and collagen III in fibroblasts (Fig. 6D-E).

Next, Dio-labeled vesicles obtained from the culture supernatant of WT and IL-37-Tg cardiomyocytes were introduced into fibroblasts pretreated with or without CPZ, a small-molecule inhibitor of the endocytic pathway. As shown in Fig. 6F and G, the uptake of vesicles in fibroblasts co-cultured with HG-treated WT cardiomyocytes was higher than that in fibroblasts co-cultured with HG-treated IL-37-Tg cardiomyocytes. In the presence of CPZ, the differential uptake of vesicles induced by IL-37 was abolished. Meanwhile, IL-37 no longer regulated collagen synthesis in CPZ-treated fibroblasts (Fig. 6H-J). Hence, these results suggested that IL-37 suppressed the profibrotic process in DCM mice by regulating either the release or the composition of cardiomyocyte-derived extracellular vesicles.

### Decreased mtDNA abundance in IL-37-Tg cardiomyocyte-derived vesicles

**Fig. 7:**
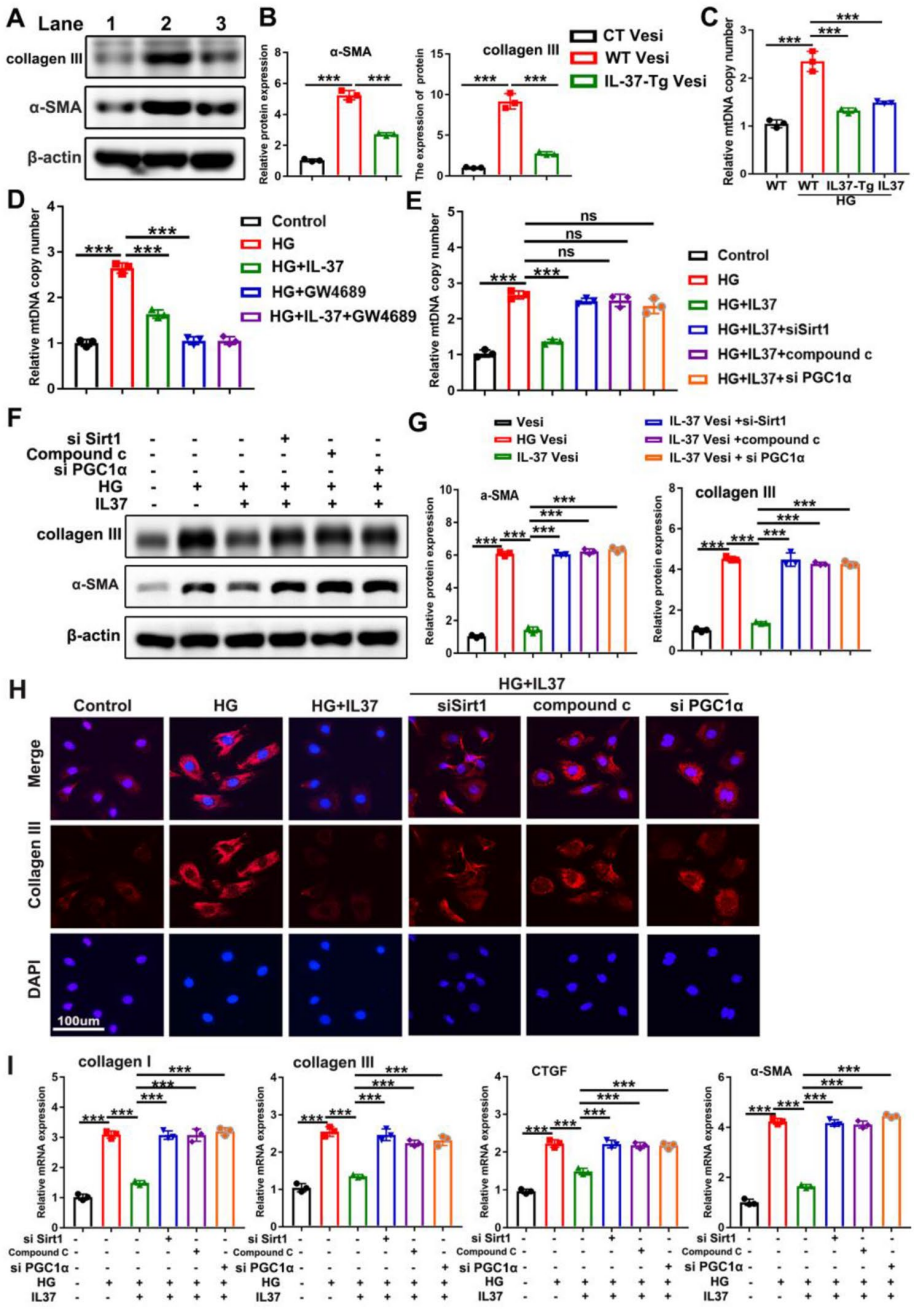
Decreased mtDNA Abundance in IL-37-Tg Cardiomyocytes-derived Vesicles. (**A** and **B**) The same number of vesicles isolated from HG-treated WT and IL-37-Tg cardiomyocytes were administered to primary cardiac fibroblasts, and the profibrotic proteins were determined. Lane 1 represents fibroblast treated with vesicles obtained from untreated WT cardiomyocytes (CT vesi); Lane 2 represents fibroblast treated with vesicles obtained from HG-treated WT cardiomyocytes (WT vesi); Lane 3 represents fibroblast treated with vesicles obtained from HG-treated IL-37-Tg cardiomyocytes (IL-37-Tg vesi). (**C**) Mitochondrial DNA content in the culture supernatant was detected by qPCR. (**D** and **E**) Cardiomyocytes, either treated with HG or not, were subjected to treatment with GW48496 (**D**), si-SIRT, compound C, si-PGC1α (**E**) in the presence or absence of IL-37. Subsequently, vesicles present in the culture supernatant were isolated and mtDNA content within them was quantified. (**F** and **G**) HG-treated or untreated cardiomyocytes were stimulated with si-SIRT, compound C or si-PGC1α in the presence of IL-37, and then vesicles in the culture supernatant were extracted and administered to primary fibroblasts for 24 h. The protein levels of collagen III and α-SMA were determined. Vesi represents fibroblasts treated with vesicles from untreated cardiomyocytes; HG Vesi represents fibroblasts treated with vesicles from HG-treated cardiomyocytes; IL-37 Vesi represents fibroblasts treated with vesicles derived from HG and IL-37-treated cardiomyocytes; “IL-37 Vesi+si-Sirt” refers to fibroblasts treated with vesicles obtained from HG-treated cardiomyocytes with SIRT1 knockdown in the presence of IL-37; “IL-37 Vesi+Compound C” refers to fibroblasts treated with vesicles derived from cardiomyocytes exposed to HG and Compound C in the presence of IL-37; “IL-37 Vesi+ si-PGC1α” refers to fibroblasts treated with vesicles derived from cardiomyocytes exposed to HG and si-PGC1α in the presence of IL-37 (**F** and **G**). Fibroblasts treated as described in H were subjected to collagen III staining (**H**) and qPCR analysis (**I**). Scale bar, 100 μm. Values are means ± SEM; *P< 0.05, **P< 0.01, ***P< 0.001. (*n* = 3 in each group at least, data were analyzed by one way ANOVA followed by Bonferroni post hoc test.)

To determine whether the enhanced activation of fibroblasts co-cultured with WT cardiomyocytes was attributed to increased vesicular uptake or selective enrichment of specific components within vesicles, vesicles isolated from both WT and IL-37-Tg cardiomyocytes were quantified, and the same amount was added to fibroblast. As shown in Supplementary Figure S7, no significant difference in vesicle uptake was observed between fibroblasts co-cultured with HG-treated WT and IL-37-Tg cardiomyocytes. However, the level of fibrosis in fibroblasts co-cultured with vesicles obtained from HG-treated WT cardiomyocytes was still higher than that in HG-treated IL-37-Tg cardiomyocytes (Fig. 7A-B). All of these findings suggested that the anti-fibrosis effect of IL-37 may be attributed to its ability to inhibit the specific enrichment of components within vesicles.

Based on this, the component in cardiomyocyte-derived vesicles that was responsible for activating cardiac fibroblasts has not been identified. Growing studies have found that mtDNA leaked from damaged mitochondria could be encapsulated in extracellular vesicles and transferred to recipient cells [32, 33]. Previously, we found that the circulating mtDNA level in the plasma of DCM patients was increased. Furthermore, we also found that the circulating mtDNA level in the plasma of DCM mice was increased, and decreased by IL-37(Supplementary Figure S8). We then quantified the mtDNA levels in vesicles derived from HG-treated WT cardiomyocytes, IL-37-Tg cardiomyocytes or IL-37-treated cardiomyocytes, and a relatively lower abundance of mtDNA in vesicles derived from both IL-37-Tg and IL-37-treated cardiomyocyte was observed when compared to those obtained from WT cardiomyocyte (Fig. 7C). Treatment with GW4869, Compound C, SIRT1-targeted siRNA or PGC-1α-targeted siRNA reversed the reduction in mtDNA abundance caused by IL-37 treatment, indicating the involvement of the SIRT1/AMPK/PGC-1α axis in IL-37-mediated inhibition of mtDNA release (Fig. 7D-E). Accordingly, the inhibitory effect of vesicles derived from IL-37-treated cardiomyocytes on collagen synthesis in fibroblasts was abolished by Compound C, or siRNA specifically targeting SIRT1 or PGC1α (Fig. 7F-G). Immunofluorescence and q-PCR further confirmed the involvement of SIRT1/AMPK/PGC-1α axis in IL-37-mediated inhibition of fibrosis (Fig. 7H-I). Our results, to a certain extent, demonstrated that IL-37 downregulates fibroblast activity by ameliorating mitochondrial damage and subsequent mtDNA release.

### MtDNA-rich vesicles depended on the TLR9 and cGAS-STING pathways to promote fibrosis

**Fig. 8:**
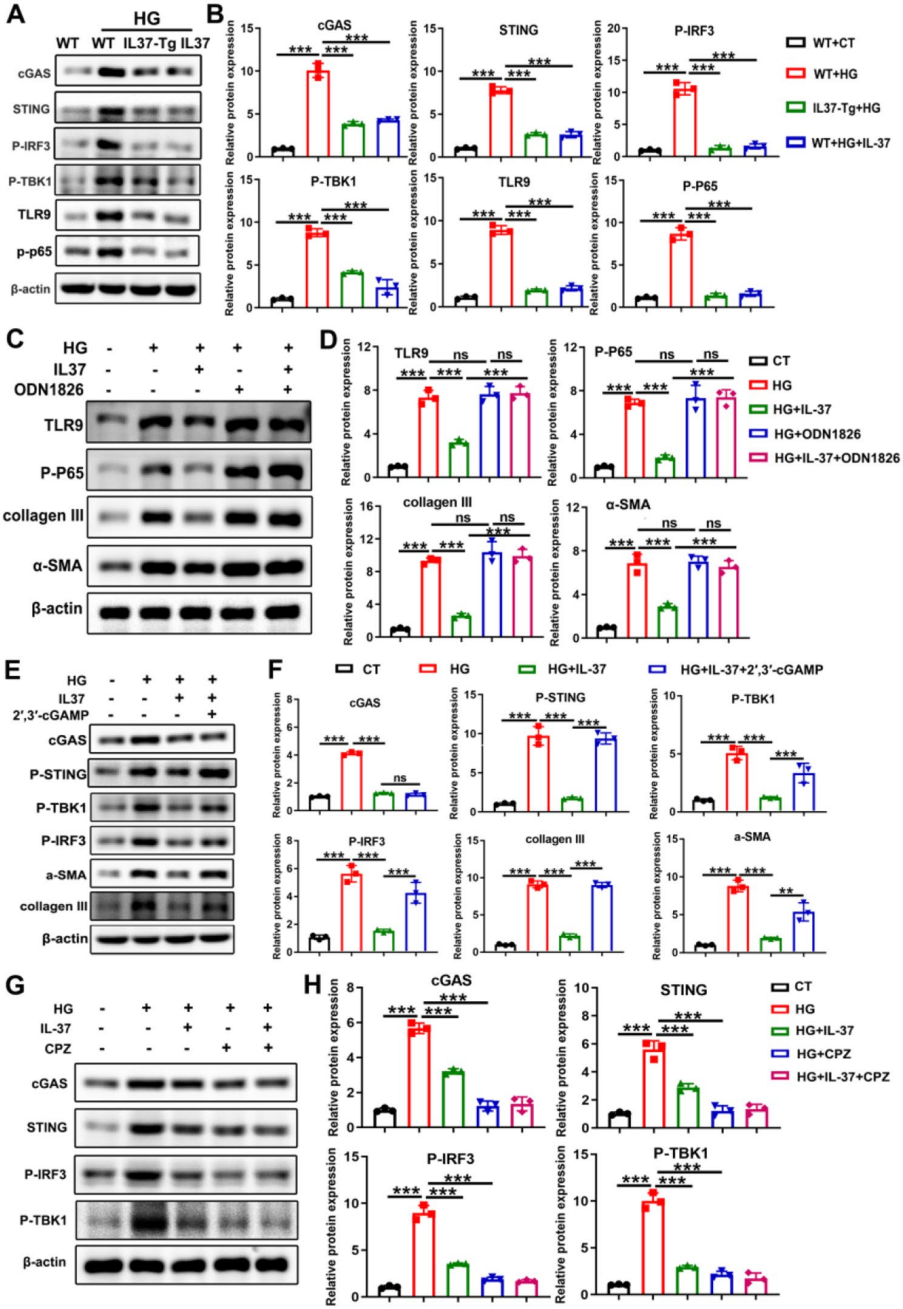
mtDNA-rich Vesicles Depended on TLR9 and cGAS-STING Pathways to Promote Fibrosis. (**A** and **B**) Primary fibroblasts were co-cultured with vesicles obtained from WT cardiomyocytes, IL-37-Tg cardiomyocytes or IL-37-treated WT cardiomyocytes in the presence or absence of HG. The activation of TLR9 and cGAS-STING pathways in fibroblasts was assessed by western blot analysis and quantified using Image J software. “WT+CT” refers to fibroblasts co-cultured with untreated WT cardiomyocyte vesicles; “WT+HG” refers to fibroblasts co-cultured with HG-treated WT cardiomyocyte vesicles; “IL-37-Tg+HG” represents fibroblasts co-cultured with HG-treated IL-37-Tg cardiomyocyte vesicles; “WT+HG+IL-37” refers to fibroblasts co-cultured with vesicles from WT cardiomyocytes exposed to HG and recombinant IL-37. (**C** and **D**) Fibroblasts were pre-treated with or without ODN1826 and then co-cultured with vesicles from cardiomyocytes in the presence or absence of HG and IL-37. The levels of indicated proteins were subsequently analyzed. (**E** and **F**) Fibroblasts, with or without cGAS silencing, were co-cultured with vesicles from cardiomyocytes exposed to HG or IL-37. The levels of p-TBK1, p-IRF3, cGAS, STING, α-SMA and collagen III were detected. (**G** and **H**) Fibroblasts pretreated with or without CPZ were co-cultured with IL-37 or HG-treated cardiomyocyte vesicles and the protein levels of cGAS, STING, p-IRF3, p- TBK1 were analyzed using western blotting. Values are means ± SEM; *P< 0.05, **P< 0.01, ***P< 0.001. (*n* = 3 in each group at least, data were analyzed by one way ANOVA followed by Bonferroni post hoc test.)

Next, we studied how mtDNA activated the fibrotic process. Previous research has demonstrated that mtDNA functions as a DAMP, capable of being recognized by the DNA sensor cGAS or TLR9 signal to initiate an immune response and fibrosis [34–36]. Therefore, the activation of cGAS and TLR9 signaling was detected. As shown in Fig. 8A-B, fibroblasts co-cultured with vesicles obtained from HG-treated cardiomyocytes exhibited elevated levels of p-TBK1, p-IRF3, p-P65, TLR9, cGAS and STING compared to those co-cultured with untreated cardiomyocyte vesicles. However, the upregulation of the above proteins was inhibited in the presence of IL-37 or upon induction of IL-37 expression. The protein levels of TLR9, p-P65, cGAS, STING, p-TBK1 and p-IRF3 showed consistent changes in the myocardium of DCM mice and IL-37 intervention groups (Supplementary Figure S9A-B). ODN1826, a TLR9 activator, eliminated the differences in the levels of TLR9 and p-P65 between fibroblasts co-cultured with IL-37-treated and untreated cardiomyocyte vesicles (Fig. 8C-D). Activating STING by 2’,3’-cGAMP in fibroblasts abrogated the difference in p-TBK1 and p-IRF3 levels caused by IL-37 treatment (Fig. 8E-F). Both ODN1826 treatment and 2’,3’-cGAMP treatment effectively nullified the inhibitory effect of IL-37 on α-SMA and collagen III expression in fibroblasts (Fig. 8C-F). In addition, pretreatment of fibroblasts with CPZ reversed the difference in cGAS-STING signaling observed between fibroblasts co-cultured with IL-37-treated and untreated cardiomyocyte vesicles (Fig. 8G-H). Finally, we also observed that the expression levels of inflammatory factors of downstream of TLR9 and cGAS-STING signaling pathways, such as IL-1β、TNF-α、IL-6 and IFN-β presented elevated in fibroblasts co-cultured with vesicles obtained from HG-treated cardiomyocytes, which was inhibited in the presence of IL-37 or upon induction of IL-37 expression(Supplementary Figure S10A). Activating STING by 2’,3’-cGAMP in fibroblast or pretreatment of fibroblasts with CPZ reversed the change of above inflammatory factors after co-cultured with vesicles obtained from HG-treated cardiomyocytes and in the presence of IL-37 (Supplementary Figure S10B-C). In short, these results indicated that cardiomyocytes treated with HG could release mtDNA-enriched vesicles, which activated the TLR9 and cGAS-STING pathways in fibroblasts, thereby promoting fibrosis and inflammation. However, IL-37 can reduce the abundance of mtDNA within vesicles, thereby alleviating adverse ventricular remodeling in DCM mice.

## Discussion

In this study, we have made several important observations. A critical finding was the upregulation of IL-37 in patients with DCM. Furthermore, IL-37 treatment or inducing IL-37 expression alleviated cardiac dysfunction and myocardial fibrosis in the hearts of diabetic mice. Mechanically, hyperglycemia impaired mitochondrial function, leading to cardiomyocyte apoptosis and the release of extracellular vesicles containing mtDNA. Fibroblasts then engulfed these mtDNA-enriched vesicles, activating TLR9 signaling and the cGAS-STING pathway, ultimately resulting in fibrosis. However, the presence of IL-37 mitigated mitochondrial injury by preserving the activity of SIRT1-AMPK-PGC1α axis, leading to a decrease in extracellular vesicle release and mtDNA abundance within vesicles. Our study demonstrated the vital role of IL-37 in DCM and established IL-37 as a potential therapeutic agent for DCM.

IL-37, a novel member of the IL-1 cytokine family with natural anti-inflammatory properties, has been reported to play a protective role in various diseases, including cardiovascular disease, tumors, rheumatoid arthritis and pulmonary fibrosis [7, 37]. In a myocardial infarction model, we showed that treatment with IL-37 significantly alleviated cardiac dysfunction and fibrosis [38]. In this study, we observed a paradoxical increase in circulating levels of IL-37 among patients with diabetes and DCM, which may indicate a compensatory response to IL-37 resistance in these pathological conditions. Furthermore, cardiomyocyte-specific IL-37 expression is closely associated with improvements in cardiac parameters and fibrosis, suggesting that IL-37 has a potential protective effect on DCM.

Dysfunctional mitochondria are the primary generators of intracellular ROS in diabetic hearts, and significantly contribute to pathological remodeling in DCM [39]. Mitochondrial functional abnormalities have been observed in various types of diabetic models and are closely associated with impaired EF and FS [40]. Furthermore, cardiac biopsies of patients with diabetes exhibit notably elevated mitochondrial ROS levels [41]. In the current study, diabetic hearts also exhibited obvious mitochondrial structural and functional abnormalities, excessive ROS production, and cardiomyocyte apoptosis, all of which were alleviated by IL-37 intervention. Multiple studies have demonstrated the critical role of the SIRT1/AMPK/PGC1α axis in maintaining mitochondrial function [42, 43]. Impairment of this axis leads to mitochondrial dysfunction, thereby exacerbating DCM development [44]. Induction of IL-37 expression increased the levels of SIRT1, p-AMPK and PGC1α, which in turn maintained mitochondrial function and subsequently suppressed cardiomyocyte apoptosis. Moreover, the mitigation of mitochondrial injury and cardiomyocyte apoptosis induced by IL-37 was abolished when SIRT1 and/or PGC1α expression was silenced or AMPK activation was inhibited. AMPK and SIRT-1 are involved in a cycle where they regulate each other in a variety of important biological functions [42]. Our results indicated that AMPK was situated downstream of SIRT1, as evidenced by the fact that silencing SIRT1 could nullify the IL-37-induced upregulation of p-AMPK levels, while inhibition of AMPK did not impact the upregulation of SIRT1. Therefore, we have identified the SIRT1/AMPK/PGC1α axis as a crucial intracellular mechanism by which IL-37 exerted its protective effects against mitochondrial dysfunction and cardiomyocyte apoptosis in DCM. IL-R8 was involved in the activation of the SIRT1-AMPK-PGC1α axis by IL-37, as demonstrated by the failure of IL-37 to activate this axis in cells with silenced IL-R8 expression (data not shown). The mechanism by which IL-R8 modulates the SIRT1-AMPK-PGC1α axis requires further investigation.

Intercellular crosstalk between cardiomyocytes and fibroblasts via paracrine signaling (including exosomes), plays a critical role in cardiac remodeling during heart failure [45]. Thus far, the precise mechanism of crosstalk between them in the cardiac fibrosis process is still poorly understood. Datta et al. demonstrated that cardiomyocyte-derived vesicles containing Hsp90 and IL-6 are transferred to fibroblasts during cardiac hypertrophy, leading to the activation of fibrotic responses in cardiac fibroblasts [46]. In addition, fibroblast-derived microRNA-enriched exosomes establish communication with cardiomyocytes, and act as a potential regulator of cardiomyocyte hypertrophy and apoptosis [47, 48]. IL-37 treatment significantly attenuated the fibrotic process both in vivo and in vitro, and this effect was abrogated by silencing SIRT1 and PGC1α or treatment with Compound C (an AMPK inhibitor), GW4869 (an exosome secretion inhibitor) and CPZ (an inhibitor of the endocytosis pathway).These results suggested that IL-37 might protect cardiomyocytes from releasing certain toxic molecule-containing extracellular vesicles (EVs), which are crucial for intercellular communication and can be transferred to fibroblasts, thereby promoting the fibrotic process [49]. One significant consequence of mitochondrial dysfunction is the release of mtDNA, which may escape from the mitochondria and leak into the cytoplasm or be encapsulated within vesicles and secreted extracellularly [49, 50]. Compared to the WT group, IL-37-Tg cardiomyocytes exhibited a significant decrease in EV amount and a relatively lower abundance of mtDNA within these vesicles. Vesicles containing mtDNA between cardiomyocytes and fibroblasts in diabetic hearts may hold potential research directions for future treatment targets.

Previous studies have demonstrated that mtDNA can trigger inflammatory cytokine production and aggravate lung fibrosis through TLR9 signaling and noncanonical cGAS-STING signaling [51]. In thoracic transverse aortic constriction induced cardiomyopathy, mtDNA is capable of inducing dilated cardiomyopathy through TLR9 signaling [22]. The augmented activation of TLR9 signaling and cGAS-STING pathway, as well as increased collagen deposition in fibroblasts co-cultured with WT cardiomyocytes, were attenuated in the presence of IL-37 and PGC1α silencing. Moreover, these disparities depended on SIRT, AMPK and PGC1α. Interestingly, the uptake of mtDNA-containing vesicles in fibroblasts co-cultured with WT cardiomyocytes seemed much higher than that in fibroblasts co-cultured with IL-37-Tg cardiomyocytes. The possibility that differences in vesicle uptake could account for variations in TLR9 and cGAS-STING signaling activation, as well as fibrosis levels, was excluded. When equal amounts of vesicles from WT and IL-37-Tg cardiomyocytes were administered to fibroblasts, the activation of TLR9 signaling and cGAS-STING pathway was still significantly higher in fibroblasts treated with vesicles derived from WT cardiomyocytes. Additionally, these fibroblasts exhibited enhanced fibrosis levels.

Several limitations of the study must be considered when interpreting the data. First, we used a diet and low-dose STZ induced mouse model of diabetes mellitus, which more resembled the clinical presentation of late stage T2D with β-cell destruction. An obvious high glucose, low insulin level and dyslipidemia were observed in our model, so just a high glucose treatment of cardiomyocytes in vitro experiment may exist a certain degree of difference with complex pathomechanisms of DCM. Second, as IL-37 is not found in mice, instead of knocking out of IL-37, we could just take the approach of IL-37 overexpression or exogenous administration to investigate its role in DCM. Third, mtDNA-enriched vesicle release from cardiomyocytes has only been demonstrated in vitro experiment, therefore, the exactly visual process of interaction between cardiomyocytes and fibroblasts in DCM warrants further investigation.

In summary, our study identified IL-37 as a key mediator in protecting against DCM by preserving mitochondrial function through the SIRT-AMPK-PGC1α axis. This led to reduced vesicle secretion in cardiomyocytes and relatively lower mtDNA abundance within these vesicles, thereby suppressing TLR9 signaling and cGAS-STING pathway-mediated profibrotic signaling in fibroblasts. These findings suggest that targeting TLR9 and the cGAS-STING pathway may be a promising approach for developing new drugs against DCM, while IL-37 could serve as a therapeutic option for treating patients with DCM.

### Electronic supplementary material

Below is the link to the electronic supplementary material.

Supplementary Material 1

## Data Availability

The data supporting the findings of this study are included in the supplemental material. The datasets used and/or analysed during the current study are available from the corresponding author upon reasonable request. No data are deposited in databases.

## Abbreviations

DCM: dDiabetic cardiomyopathy
DAMPs: damage-associated molecular patterns
mtDNA: mitochondrial DNA
CM: cardiomyocyte
CF: fibroblast
TEM: Transmission electron microscopy
ΔΨm: mitochondrial membrane potential
TC: total cholesterol
TG: triacylglycerol
EF: ejection fraction
FS: fractional shortening
LVIDIs: LV internal dimension index diastole
LVEDD: LV end-diastolic dimension
LVEDV: LV end-diastolic volume
