# Supplementary material for: IL-37 ameliorates myocardial fibrosis by regulating mtDNA-enriched vesicle release in diabetic cardiomyopathy mice
Source: J Transl Med. 2024 May 24;22:494. doi: 10.1186/s12967-024-05250-3 (PMC11127460; doi:10.1186/s12967-024-05250-3)
Supplement: Supplementary file 1 — Supplementary Material 1 [file 12967_2024_5250_MOESM1_ESM.docx]

Supplementary materials

| **Variable** | **NM (n=22)** | **DM (n=20)** | **DCM (n=27)** | ***P* value** |
| --- | --- | --- | --- | --- |
| Age (years) | 60.7±2.4 | 61.3±2.1 | 72.1±1.4 | ＜0.001 |
| Gender (male), n(%) | 8 (36.4) | 10 (50.0) | 10 (37.0) | 0.595 |
| BMI (kg/m^2^) | 25.3±0.8 | 26.0±1.0 | 25.9±0.7 | 0.790 |
| proBNP (pg/mL) | 61.3±11.4 | 79.3±19.6 | 351.8±89.8 | 0.001 |
| cTnT (ng/mL) | 0.007±0.001 | 0.011±0.002 | 0.023±0.007 | 0.046 |
| IL-37 (pg/mL) | 2.8±0.4 | 2.7±0.3 | 16.8±1.5 | ＜0.001 |
| UA (μmol/L) | 320.8±22.0 | 357.7±30.0 | 325.9±16.3 | 0.482 |
| CRE (mmol/L) | 72.5±4.1 | 89.8±9.3 | 75.9±5.4 | 0.156 |
| ALT (U/L) | 23.2±3.9 | 24.9±3.4 | 17.3±2.2 | 0.187 |
| AST (U/L) | 21.2±1.7 | 20.4±1.3 | 18.3±1.3 | 0.301 |
| HbAlc (%) | 5.5±0.1 | 8.3±0.5 | 7.8±0.3 | ＜0.001 |
| FBG (mmol/L) | 5.4±0.1 | 9.2±1.3 | 7.8±0.6 | 0.005 |
| LDL-C (mmol/L) | 2.6±0.2 | 2.4±0.2 | 2.3±0.2 | 0.515 |
| Total cholesterol (mmol/L) | 4.3±0.2 | 4.5±0.3 | 3.9±0.2 | 0.226 |
| Triglycerides (mmol/L) | 1.6±0.1 | 2.4±0.3 | 1.5±0.1 | 0.012 |
| HDL-C (mmol/L) | 1.3±0.1 | 1.0±0.1 | 1.1±0.1 | 0.102 |
| CRP (mg/L) | 3.3±1.1 | 2.9±0.6 | 5.5±1.8 | 0.339 |
| LAD (mm) | 36.1±0.8 | 38.6±0.9 | 40.2±0.6 | 0.001 |
| LVIDd (mm) | 49.0±1.1 | 47.1±1.0 | 49.2±1.0 | 0.293 |
| LVIDs (mm) | 31.6±0.9 | 31.3±0.7 | 33.7±1.0 | 0.117 |
| IVSd (mm) | 10.1±0.3 | 10.6±0.3 | 11.5±0.3 | 0.003 |
| LVPWd(mm) | 9.2±0.2 | 9.5±0.2 | 10.0±0.2 | 0.017 |
| LVEF (%) | 64.1±1.1 | 62.0±1.1 | 57.7±1.7 | 0.006 |
| E/A ratio | 1.0±0.03 | 0.9±0.03 | 0.7±0.38 | ＜0.001 |
| E/e' ratio | 6.9±0.4 | 6.9±0.2 | 9.8±0.7 | ＜0.001 |

Table1. The general characteristics of study population

Data presented as mean±SEM or n (%), one way ANOVA followed by Bonferroni post hoc test or chi-square test was used to check for normal distributions of data. BMI, body mass index; BNP, brain natriuretic peptide; cTnT, cardiac troponin T; IL-37, interleukin 37; UA, uric acid; CRE, creatinine; ALT, alanine aminotransferase; AST, aspartate aminotransferase; HbAlc, glycosylated hemoglobin Alc; FBG, fasting blood glucose; LDL-C, low-density lipoprotein cholesterol, HDL-C, high-density lipoprotein cholesterol; CRP, C-reactive protein; LAD, left atrial diameter; LVIDd, left ventricular internal diameter at end-diastole; LVIDs, left ventricular internal diameter at end-systole; IVSd, interventricular septum thickness at end-diastole; LVPWd, left ventricular posterior wall thickness at end-diastole; LVEF, left ventricular ejection fraction; E, passive transmitral LV inflow velocity; A, late transmitral LV inflow during left atrial contraction; e', tissue Doppler imaging velocity of the medial mitral annulus during passive filling.


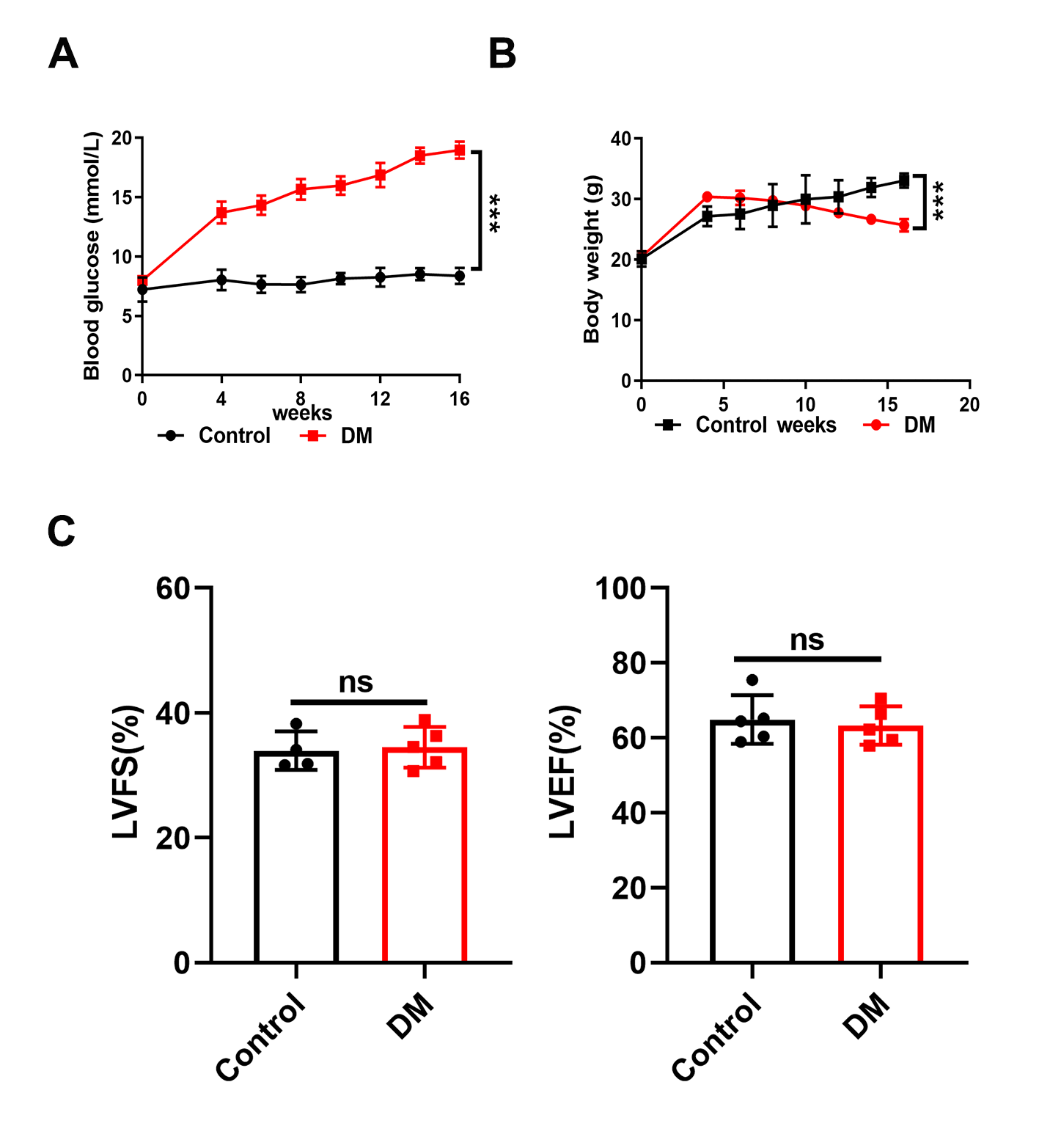


Figure S1

(A) The blood glucose levels in both control and diabetic mice. (B) Body weight changes were measured in both control and diabetic mice. (C and D) Left ventricular fractional shortening (LVFS) and left ventricular ejection fraction (LVEF) in both control and diabetic mice were also evaluated. Values are means ± SEM; *P< 0.05, **P< 0.01, ***P< 0.001. (n = 6 in each group, data were analyzed by one way ANOVA followed by Bonferroni post hoc test or unpaired Student t-test.)


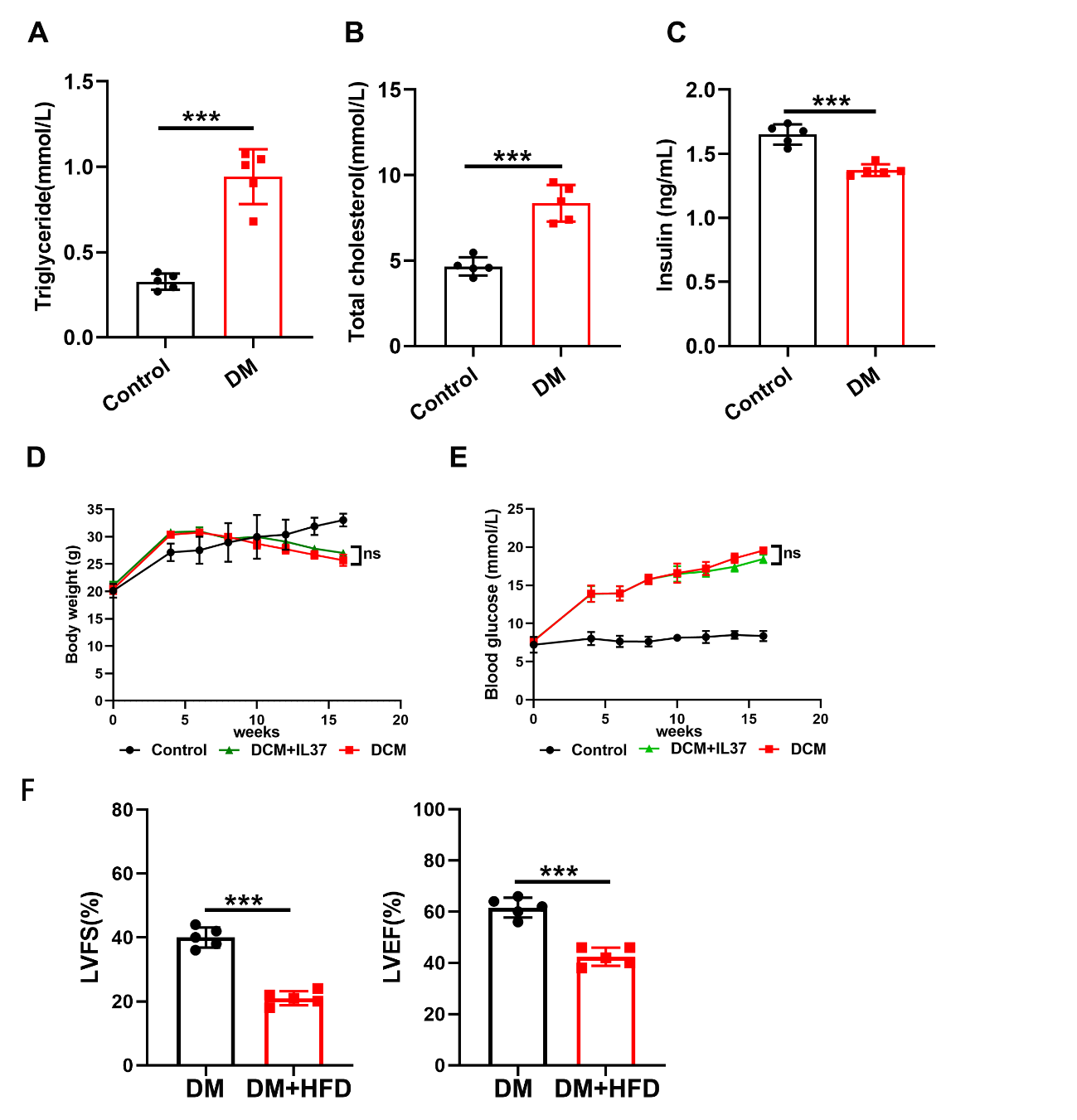


Figure S2 (A-C) The serum levels of triglyceride, cholesterol, and insulin in both control and diabetic mice were measured. (D) The body weight changes were analyzed among the indicated groups. (E) Blood glucose levels were measured in the indicated groups. (F) LVFS and LVEF measurements were obtained. Values are means ± SEM; *P< 0.05, **P< 0.01, ***P< 0.001. (n = 6 in each group, data were analyzed by one way ANOVA followed by Bonferroni post hoc test or unpaired Student t-test.)


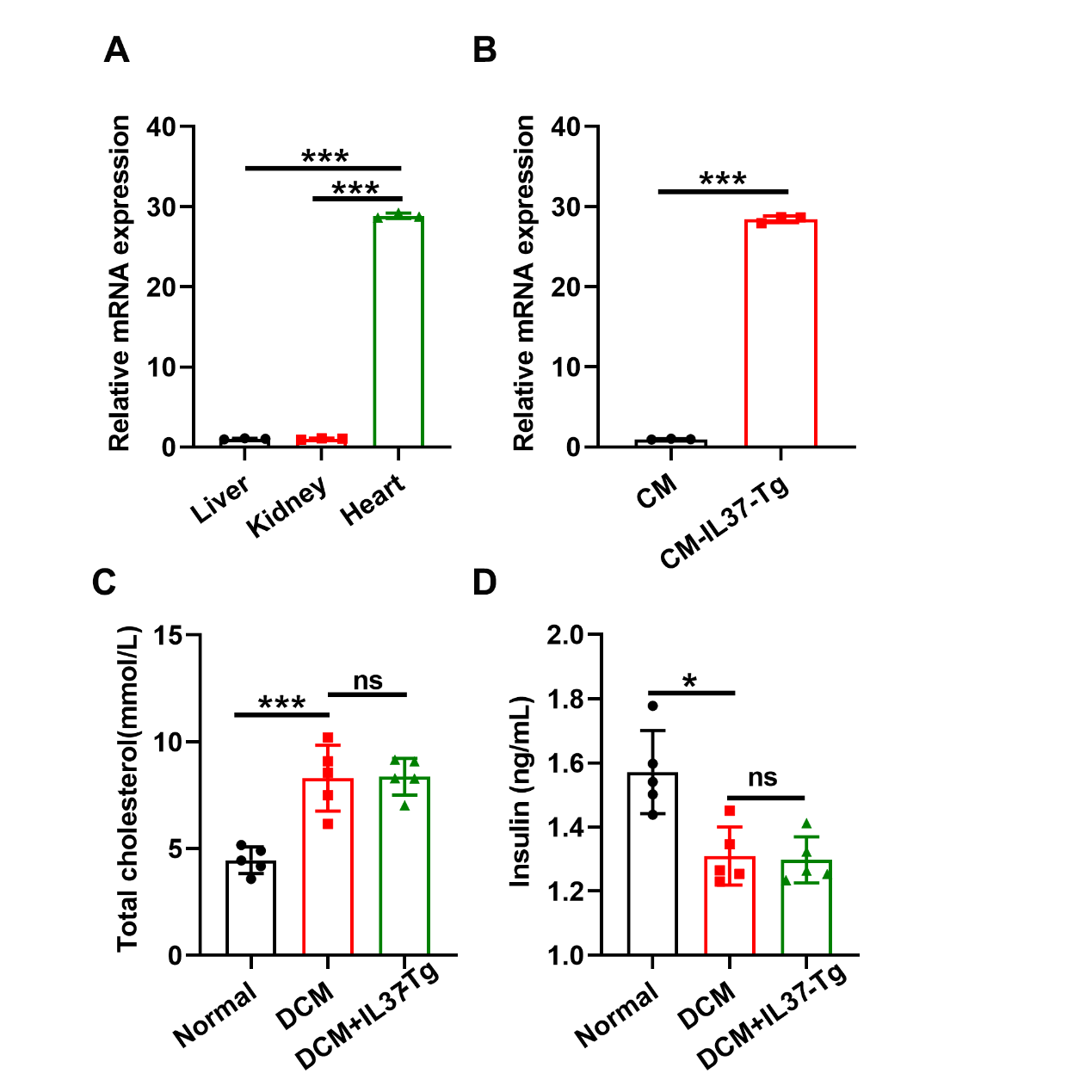


Figure S3 (A)The mRNA level of IL37 in different tissues. (B) The expression of IL-37 in WT and IL37-Tg cardiomyocyte. (C and D) The serum levels of cholesterol and insulin in the indicated groups were measured. Values are means ± SEM; *P< 0.05, **P< 0.01, ***P< 0.001. (n = 6 in each group, data were analyzed by one way ANOVA followed by Bonferroni post hoc test or unpaired Student t-test.)


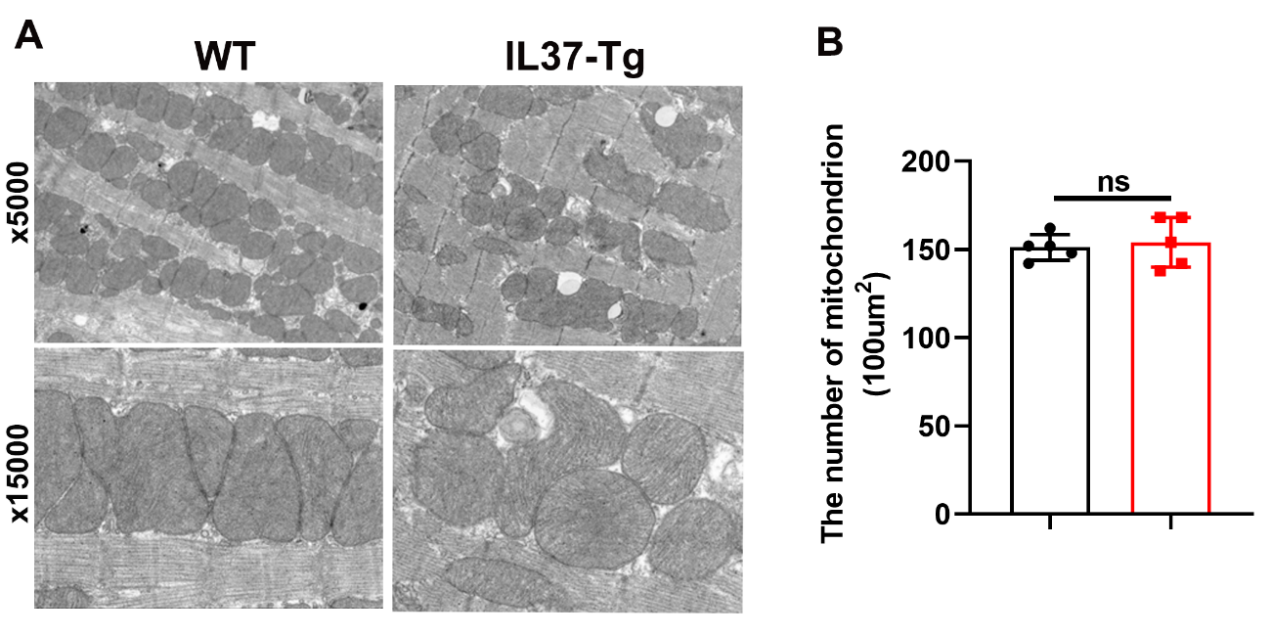


Figure S4 (A)The representative electron microscopy (EM) images of mitochondria in WT and IL-37-Tg mice were presented. (B) Quantification of mitochondrial abundance in panel A was shown. Values are means ± SEM; *P< 0.05, **P< 0.01, ***P< 0.001. (n = 6 in each group, data were analyzed by unpaired Student t-test.)


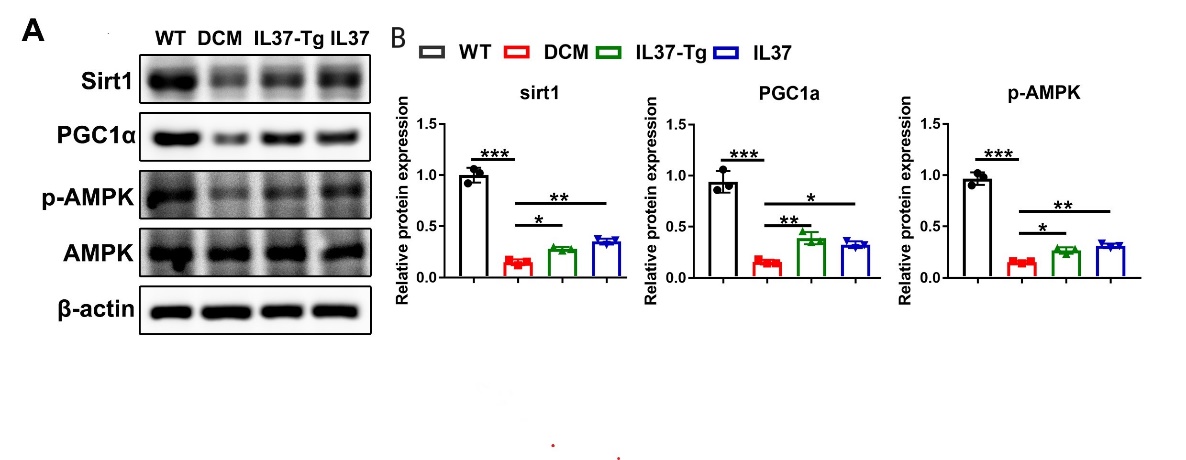


Figure S5 (A) The protein levels of SIRT1, PGC1α, p-AMPK and AMPK were assessed in the myocardium of mice. (B) Quantitative densitometric analysis of SIRT1, PGC1α, p-AMPK and AMPK with β-actin as an internal standard. Values are means ± SEM; *P< 0.05, **P< 0.01, ***P< 0.001. (n = 6 in each group, data were analyzed by one way ANOVA followed by Bonferroni post hoc test.)


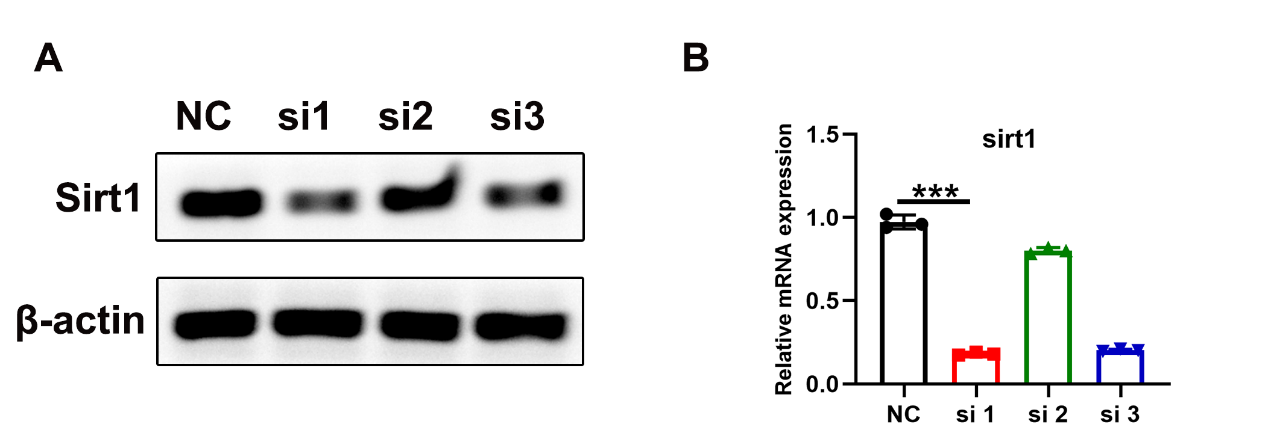


Figure S6 (A and B) The efficiency of sirt1 silencing was validated by Western blot and q-PCR analyses. Values are means ± SEM; *P< 0.05, **P< 0.01, ***P< 0.001, (n = 3 in each group at least, data were analyzed by one way ANOVA followed by Bonferroni post hoc test.)


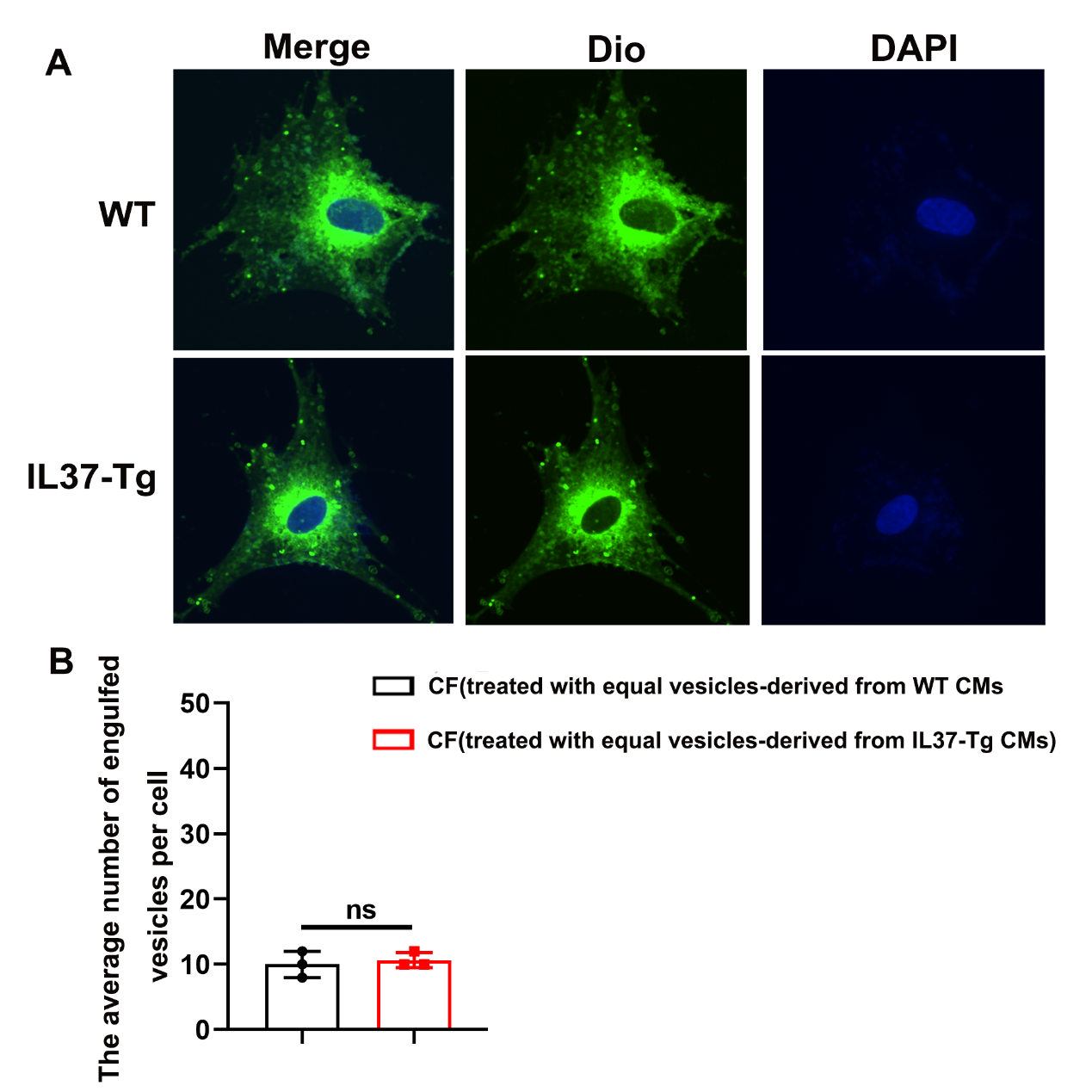


Figure S7 (A) The representative images of engulfment vesicles were presented. (B)The quantification of engulfed vesicles was assessed in both experimental groups. Values are means ± SEM; *P< 0.05, **P< 0.01, ***P< 0.001. (n = 3 in each group at least, data were analyzed by unpaired Student t-test.)


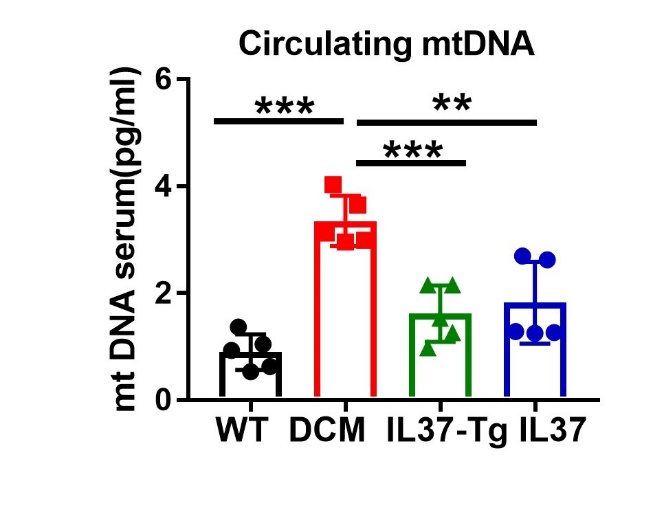


Figure S8 The circulating mtDNA level in serum samples of mice, as measured by RT-PCR. Values are means ± SEM; *P< 0.05, **P< 0.01, ***P< 0.001. (n = 6 in each group, data were analyzed by one way ANOVA followed by Bonferroni post hoc test.)


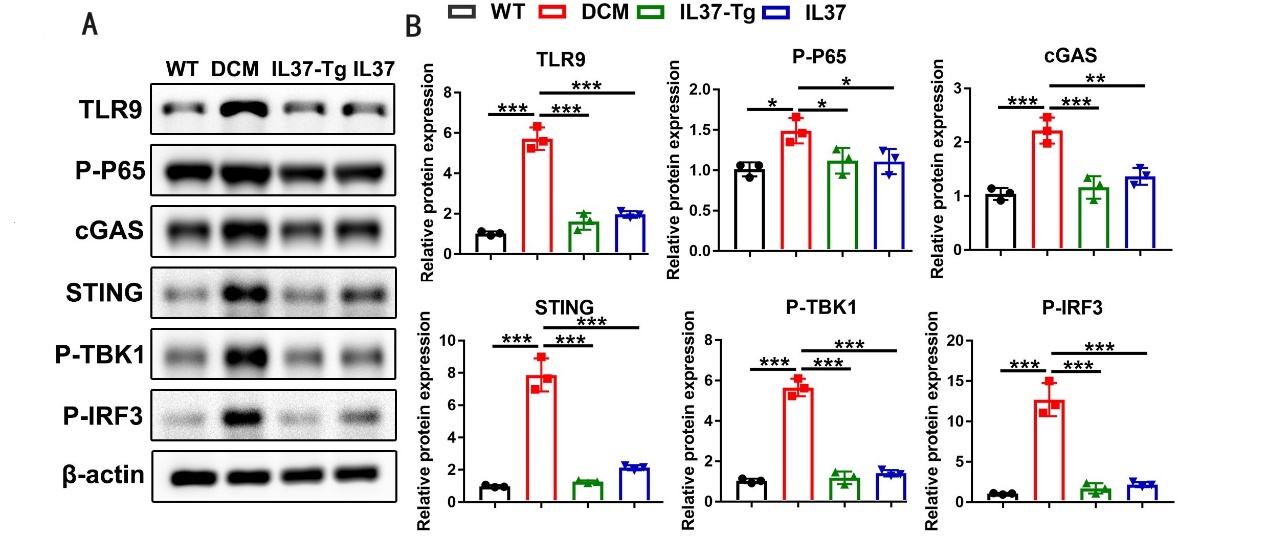


Figure S9 (A) The protein levels of TLR9, p-P65, cGAS, STING and p-TBK1, p-IRF3 were assessed in the myocardium of mice. (B) Quantitative densitometric analysis of TLR9, p-P65, cGAS, STING and p-TBK1, p-IRF3 with β-actin as an internal standard. Values are means ± SEM; *P< 0.05, **P< 0.01, ***P< 0.001vs normal group; #P< 0.05, ## P< 0.01, ### P< 0.001 vs DCM group. (n = 6 in each group, data were analyzed by one way ANOVA followed by Bonferroni post hoc test.)


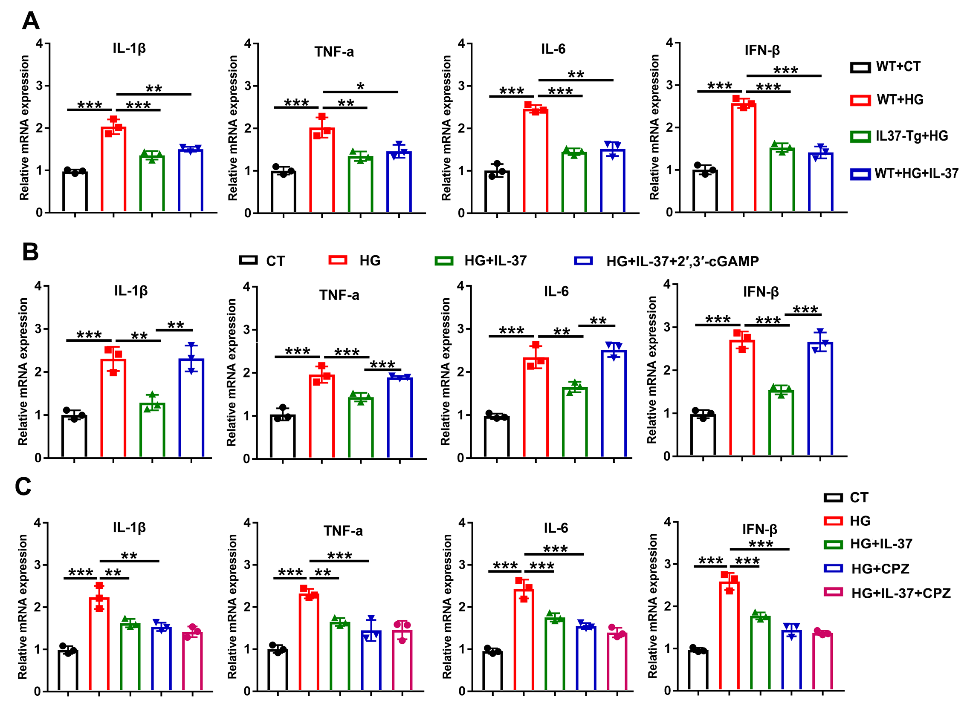


Figure S10 The mRNA level of IL-1β、TNF-α、IL-6 and IFN-β in fibroblasts after

treated with vesicles obtained from cardiomyocytes in the presence or absence of IL-37 and HG (A), and intervened by 2’,3’-cGAMP in fibroblast or pretreatment of fibroblasts with CPZ (B-C). “WT+CT” refers to fibroblasts co-cultured with untreated WT cardiomyocyte vesicles; “WT+HG” refers to fibroblasts co-cultured with HG-treated WT cardiomyocyte vesicles; “IL-37-Tg+HG” represents fibroblasts co-cultured with HG-treated IL-37-Tg cardiomyocyte vesicles; “WT+HG+IL-37” refers to fibroblasts co-cultured with vesicles from WT cardiomyocytes exposed to HG and recombinant IL-37. Values are means ± SEM; **P< 0.01, ***P< 0.001. (n = 3 in each group at least, data were analyzed by one way ANOVA followed by Bonferroni post hoc test.)

Table 2. Antibodies used in this study


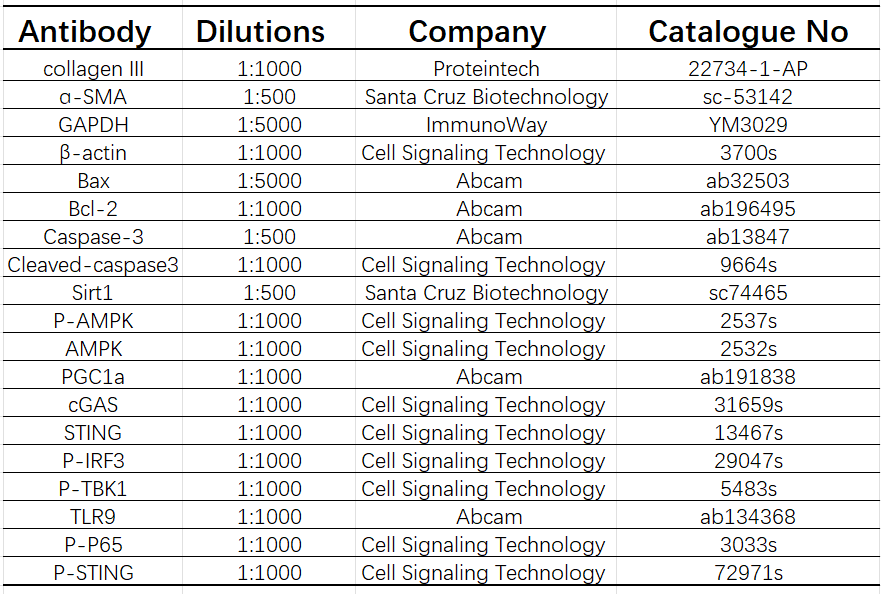


Table 3 PCR primer.

| **Genes** | **Forward (5’-3’)** | **Reverse (3’-5’)** |
| --- | --- | --- |
| CTGF | TGCGAAGCTGACCTGGAGGAAA | CCGCAGAACTTAGCCCTGTATG |
| ANP | TACAGTGCGGTGTCCAACACAG | TGCTTCCTCAGTCTGCTCACTC |
| BNP | TCCTAGCCAGTCTCCAGAGCAA | GGTCCTTCAAGAGCTGTCTCTG |
| Collagen I | CCTCAGGGTATTGCTGGACAAC | CAGAAGGACCTTGTTTGCCAGG |
| Collagen III | GACCAAAAGGTGATGCTGGACAG | CAAGACCTCGTGCTCCAGTTAG |
| ɑ-SMA | TGCTGACAGAGGCACCACTGAA | CAGTTGTACGTCCAGAGGCATAG |
| TFAM | GAGGCAAAGGATGATTCGGCTC | CGAATCCTATCATCTTTAGCAAGC |
| NRF1 | GGCAACAGTAGCCACATTGGCT | GTCTGGATGGTCATTTCACCGC |
| UCP2  mMito  mB2M  hMito  hB2M  IL-1β  TNF-α  IL-6  IFN-β  GAPDH | TAAAGGTCCGCTTCCAGGCTCA  CGTACACCCTCTAACCTAGAGAAGG  GTGGTGCCAGCAGAGACTTA  CACTTTCCACACAGACATCA  TGTTCCTGCTGGGTAGCTCT  GGGCCTCAAAGGAAAGAATC  GAACTGGCAGAAGAGGCACT  AGTTGCCTTCTTGGGACTGA  TGAATGGAAAGATCAACCTCACCTA  AACTTTGGCATTGTGGAAGG | ACGGGCAACATTGGGAGAAGTC  GGTTTTAAGTCTTACGCAATTTCC  GGACAGTGGGTAGGGAACTG TGGTTAGGCTGGTGTTAGGG  CCTCCATGATGCTGCTTACA  TACCAGTTGGGGAACTCTGC  AGGGTCTGGGCCATAGAAC  TCCACGATTTCCCAGAGAAC  CTCTTCTGCATCTTCTCCGTCA  ACACATTGGGGGTAGGAACA |
